# Probiotic Properties of *Lactobacillus helveticus* and *Lactobacillus plantarum* Isolated from Traditional Pakistani Yoghurt

**DOI:** 10.1155/2020/8889198

**Published:** 2020-12-24

**Authors:** Mahreen Ul Hassan, Hina Nayab, Farheen Shafique, Mike P. Williamson, Taghreed Saud Almansouri, Noreen Asim, Nuzhat Shafi, Safira Attacha, Madiha Khalid, Nasir Ali, Nazia Akbar

**Affiliations:** ^1^Department of Microbiology, Shaheed Benazir Bhutto Women University, Peshawar 25000, Pakistan; ^2^Department of Molecular Biology and Biotechnology, University of Sheffield, Sheffield, S10 2TN, UK; ^3^Institute of Biological Sciences, Sarhad University of Science and Information Technology, Peshawar 25000, Pakistan; ^4^Department of Biomedical Science, University of Sheffield, S10 2TN, Sheffield, UK; ^5^Department of Neuroscience (SITraN), University of Sheffield, S10 2HQ, Sheffield, UK; ^6^Department of Applied Medical Science (Medical Laboratory), King Abdulaziz University, Jeddah, Saudi Arabia; ^7^Division of Genomics and Bioinformatics, Institute of Biotechnology and Genetic Engineering, The University of Agriculture, Peshawar 25000, Pakistan; ^8^Department of Zoology, University of Azad Jammu and Kashmir, Muzaffarabad 13100, Pakistan; ^9^Department of Biotechnology, Women University of Azad Kashmir, Bagh 12500, Pakistan; ^10^Department of Microbiology, Abbottabad University of Science and Technology, Abbottabad 22010, Pakistan; ^11^Centre of Human Genetics, Hazara University Manshera, Hazara 21351, Pakistan

## Abstract

Probiotic bacteria are of utmost importance owing to their extensive utilisation in dairy products and in the prevention of various intestinal diseases. The objective of this study was to assess the probiotic properties of bacteriocin-producing isolates of *Lactobacillus helveticus* and *Lactobacillus plantarum* isolated from traditional Pakistani yoghurt. In this study, ten bacteriocin-producing isolates were selected to screen for the probiotic property. The isolates showed resistance to acidic pH (6-6.5), bile salt (0.01-1%), and 1-7% NaCl salt and showed good growth at acidic pH and antibacterial activity against ten different foodborne pathogens. Interestingly, these isolates were proved to be effective against *Actinobacter baumannii* but least effective against *Klebsiella pneumoniae* and *Pseudomonas aeruginosa*. A few isolates were found to be resistant to some antibiotics like vancomycim, gentamycin, erythromycin, streptomycin, and clindamycin. Our results provide strong evidence in favour of traditional Pakistani yoghurts as a potential source of bacteriocin-producing bacteria with an added benefit of the probiotic property. Specifically, LBh5 was considered a good probiotic isolate as compared to other isolates used in the study. Further extensive research should be done on isolation and characterisation of probiotic isolates from local fermented foods, and then, these isolates should be used in the development of probiotic enriched food supplements in Pakistan.

## 1. Introduction

Probiotic is a well-known term used for “live microorganisms” when added as supplements in food, which provide many health benefits. In the food industry, lactic acid bacteria (LAB) are the most promising group of bacteria that have been consumed fearlessly as they are generally regarded as safe (GRAS) [1]. In fact, most of them are natural inhabitants of dairy products like cheese and yoghurt and considered native microflora in dairy products [2, 3]. They are present naturally or added purposely to food products for enhancing their flavour and aroma and for the maintenance of human gut microflora. Searching fermented food items for probiotics has been a very practical and convenient approach for preservation as it does not invite objections against chemical additives being a part of the intake [4]. The present study includes *Lactobacillus*, the probiotic genus popular for many medical applications such as decreasing enteric infections, intestinal tumors, and cholesterol levels, folate production, treating cardiovascular diseases, metabolic disorders, lactose intolerance, and boosting the immune system [5–9]. Moreover, such bacteria release anticarcinogenic and antimicrobial substances in addition to organic acids such as lactic acid, benzoic acid, and acetic acid [5, 10]. The antimicrobial peptides known as bacteriocins are also defining characteristics of these bacteria, which efficiently inhibit the growth of certain foodborne pathogens and spoilage bacteria [11]. Among lactobacilli, *Lactobacillus plantarum* and *Lactobacillus helveticus* are two probiotics commonly used as a starter culture in several fermentations, especially in the fermentation of dairy products [4]. *Lactobacillus helveticus* shows profound proteolytic activity and is also reported as a good inhibitor of angiotensin-converting enzyme, which has a role in alleviating hypertension. *Lactobacillus plantarum* is an indigenous gut inhabitant and therefore is considered more compatible with the gut environment [12, 13].

The antimicrobial ability maintained by a probiotic is attributed to the production of organic acids (acetic acid and lactic acid), protein metabolites, hydrogen peroxide, cyclic dipeptides, enzymatic effects, and certain antimicrobial peptide-bacteriocins [14, 15]. Lately, isolation and characterisation of bacteriocins are of interest to microbiologists as they could be a better candidate to replace antibiotics and synthetic preservatives [4]. In the past decades, many *Lactobacillus* species were reported for their probiotic potential and exhibited antagonistic activity against various microbes, but very few studies have been done on bacteriocin-producing potential probiotic strains isolated from a dairy product.

Isolating potential probiotic strains has been practiced for centuries and is still of interest because of its industrial and medicinal value [16]. Probiotic strains must have the ability to tolerate the stress environment contained in the gastrointestinal system. Their viability in harsh conditions like the presence of bile, gastric juices, and NaCl, low pH in the stomach, adhesion to the intestinal lining, and antibiotics contributes to their efficacy [8, 17]. Their resistance to environmental stress and their bacteriocin producing ability would aid the food industry in establishing safe preservation strategies. In Pakistan, yoghurt is a famous dairy product obtained by fermentation of milk. It is consumed daily by a large population as it possesses nutritional value; plus, it is a significant source of beneficial bacteria [17]. Yoghurt from different countries has been investigated for the isolation of potential probiotics. Literature shows that a distinctive microbiome has also been reported from yoghurt of different cities of Pakistan, such as Lahore, Faisalabad, Karachi, Islamabad, and Peshawar. These isolates include *Lactobacillus bulgaricus*, *Lactobacillus casei*, *Lactobacillus acidophilus*, *Lactobacillus salivarius*, and *Leuconostoc mesenteroides* CYG362 [18, 19] from Lahore; *L. acidophilus*, *Lactobacillus delbrueckii* subsp. *bulgaricus*, *Lactobacillus paracasei* subsp. *paracasei*, *L. bulgaricus*, *L. delbrueckii*, *L. plantarum*, *Lactobacillus fermentum*, *Lactobacillus rhamnosus*, and *Streptococcus thermophilus* from Faisalabad [20–24]; *L. bulgaricus* from Islamabad [25]; *Pediococcus pentosaccus*, *L. delbrueckii*, *L. plantarum*, *L. helveticus*, and *Pediococcus acidilactici* from Peshawar; and *Lactobacillus curvatus* KIBGE-IB44 from Karachi [26, 27]. These microbes are also included in commercial probiotic yoghurt in Pakistan [28]. In Pakistan, *L. helveticus* and *L. plantarum* are rarely isolated from yoghurt as compared to other species; thus, their probiotic potential is barely determined.

In our previous study [29], we isolated bacteriocin from two lactobacilli, *L. plantarum* and *L. helveticus*, which effectively inhibited foodborne pathogens. In this study, we extended our research and evaluated the probiotic potential of bacteriocin-producing species, as very few bacteriocin producing probiotic species were described earlier. This study provides potent probiotics that could be used in food preservation and storage strategies to prevent certain spoilage bacteria and thus reduces the economic loss experienced by the food industry.

### 1.1. Contributions

Our contributions in this study are as follows:

- In an earlier study, an analysis of the bacteriocin properties of *Lactobacillus* species (isolated from traditional yoghurt) was done. Among different isolates, *Lactobacillus* species *L. helveticus* and *L. plantarum* were involved in the production of bacteriocins. In the current study, these two species were evaluated for probiotic properties. The comparative analysis of probiotic properties on both species showed that *L. helveticus* was the most suitable probiotic species as compared to *L. plantarum* against foodborne pathogens
- Among these two species, *L. helveticus* was seldom found in dairy products. It was the first time that these bacteriocin-producing isolates have been isolated from a yoghurt sample from Peshawar KPK while *L. plantarum* has been isolated a few times from dairy products. These isolates could further be subjected to various in vivo approaches to find the actual target of these isolates. Furthermore, these isolates could be assessed for the production of the unusual proteolytic spectrum and other inhibitors
- The remarkable probiotic properties of these two bacteriocin-producing species and the comparison of *L. helveticus* and *L. plantarum* make this study distinctive and interesting for readers

## 2. Materials and Methods

### 2.1. Bacterial Species Used in This Study

Ten clinically identified foodborne pathogens used in this study were ordered from the Bacteriology Lab, Department of Microbiology at the Faculty of Sciences, Punjab University, Pakistan. The included species used in this study are *Acinetobacter baumanni*, *Bacillus subtilis*, *Enterococcus faecium* DO, *Escherichia coli*, *Klebsiella pneumoniae*, *Methicillin-resistant Staphylococcus aureus* (MRSA), *Pseudomonas aeruginosa*, *Salmonella paratyphi A*, *Staphylococcus aureus*, and *Streptococcus pyogenes.* The same bacterial isolates were used in a previous study by Hassan et al. [29].

### 2.2. Isolation, Screening, and Identification of Bacteriocin-Synthesising Species

The bacteriocin-producing isolates were obtained from screening 50 traditional Pakistani yoghurt samples used in the previous study conducted by Hassan et al. [29].

### 2.3. Growth Rate Study

A starter culture of 1 mL of a 24 hrs old culture of *Lactobacillus* bacterial isolates was inoculated in 100 mL of nutrient broth and MRS broth (Merck, Darmstadt, Germany) in an anaerobic jar (BD GasPak®100 System, Becton Dickinson®, USA) with a fresh Thermo Scientific®Oxoid®AnaeroGen®.2.5 L sachet. The anaerobic jar was in a New Brunswick™ Excella®E24 Incubator Shaker at 37°C at 100 rpm [30]. Bacterial growth was monitored spectrophotometrically at 600 nm (A_600_) at 0, 2, 4, 6, 8, 10, 12, and 24 hrs. The measurements were carried out using an Agilent Cary 5000 Ultraviolet-to-Visibleto-Near-Infrared (UV-Vis-NIR) spectrophotometer (Agilent Technologies, Petaling Jaya, Malaysia) [31].

### 2.4. Evaluation of Probiotic Properties

To evaluate the probiotic potential of bacteriocin-producing isolates, the following properties were considered: tolerance to low pH, bile salts , NaCl, antibiotic susceptibility, antimicrobial activity, and response to gastroduodenal stimuli.

#### 2.4.1. Low pH Tolerance

A 1 mL bacterial culture was inoculated into eight tubes, each containing 9 mL MRS broth adjusted to varying pH ranges from 1 to 8. After 4, 6, 12, and 24 hours of incubation at 37°C, the growth rate was calculated by considering the optical density (OD) values measured at 600 nm.

Uninoculated media was taken as a negative control.

#### 2.4.2. NaCl Tolerance

Isolates were drawn in MRS broth having different NaCl concentrations ranging from 1 to 7%, incubated at 37°C for 4, 6, 12, and 24 hrs at 37°C. Growth was determined by measuring the optical density of the broth at 600 nm. A tube without NaCl was run as a negative control.

#### 2.4.3. Bile Salt Tolerance

MRS broth was supplemented with different concentrations of bile (Ox-gall): 0%, 0.05%, 0.1%, 0.3%, 0.6%, and 1%, and was inoculated with lactobacilli to investigate bile salt tolerance. ODs measured at 600 nm were used to measure cell growth after 0, 2, 4, 6, 12, and 24 hrs at 37°C.

Negative control was also run.

#### 2.4.4. Antibiotic Susceptibility Test

The agar well diffusion method was used to test antibiotic susceptibility against frequently used antibiotics. The antibiotics which were used in this study against all ten isolates with the same concentrations are ampicillin (Amp) (2 mg/L), gentamycin (Gen) (16 mg/L), erythromycin (Ery) (1 mg/L), and clindamycin (CLI) (4 mg/L). The concentration of kanamycin used for the *L. plantarum* isolates was 64 mg/L and for the *L. helveticus* isolates 16 mg/L. The two antibiotics tetracycline (Tet) at 32 mg/L and chloramphenicol (CL) at 8 mg/L were only tested against *L. plantarum* isolates. The two antibiotics streptomycin (SM) at 16 mg/L and vancomycin (Van) at 2 mg/L were only tested against *L. helveticus* isolates. The stock solution of the antibiotics was made in distilled water according to guidelines from the Clinical and Laboratory Standards Institute (CLSI) and the European Food Safety Authority (EFSA) [32, 33]. Distilled water was run as a control.

#### 2.4.5. Antibacterial Activity

A cell suspension of the *Lactobacillus* species was prepared in MRS broth by comparing the turbidity with 0.5 McFarland solution. Test microorganisms were cultured in nutrient broth and swabbed onto Muller Hinton Agar (MHA) plates (Merck, Darmstadt, Germany). Isolates were incubated for 24 hrs and then centrifuged at 12,000 × g for 10 min, and 50 *μ*L of supernatant was loaded in the well made on MHA and incubated at 37°C for 24 hrs. The antibacterial activity was determined against both gram-negative and gram-positive pathogens.

#### 2.4.6. Response to Stomach-Duodenal Stimulus

The response of the tested LAB to the stomach-duodenal stimulus was evaluated in vitro by the method described in Pinto et al. [34]. It was performed on overnight bacterial culture 10-fold dilutions to determine the OD_600_ and cell number of each dilution. Bacterial survival was determined by measuring the OD_600_ at 0, 2, 4, 6, 8, 10, 12, and 24 hrs [35].

#### 2.4.7. Arginine Hydrolysis Test

To perform the arginine hydrolysis test, MRS broth (without glucose and meat extract, supplemented with 0.3% arginine and 0.2% sodium citrate) was used [36].

### 2.5. Statistical Analysis

Statistical tools Pearson correlation and two-way ANOVA were applied to analyse the data. R program version 1.3.959, Graph Pad Prism version 8.4.3, and MS Excel 16.0 were used to calculate statistics.

## 3. Results and Discussion

### 3.1. Isolation and Identification of *Lactobacillus* spp. from Traditional Yoghurts

Bacteriocin-producing isolates were collected from yoghurt. From 12 different isolates, five isolates were identified as *L. helveticus*, and of the rest five were identified as *L. plantarum* on the basis of their colony morphology and various biochemical tests, and the remaining two isolates were identified as *E. coli* and *Enterococcus faecium.* The *L. helveticus* isolates were named LBh1, LBh2, LBh3, LBh4, and LBh5, and the *L. plantarum* isolates were named LBp1, LBp2, LBp3, LBp4, and LBp5.

### 3.2. Growth Rate Study

**Figure 1:**
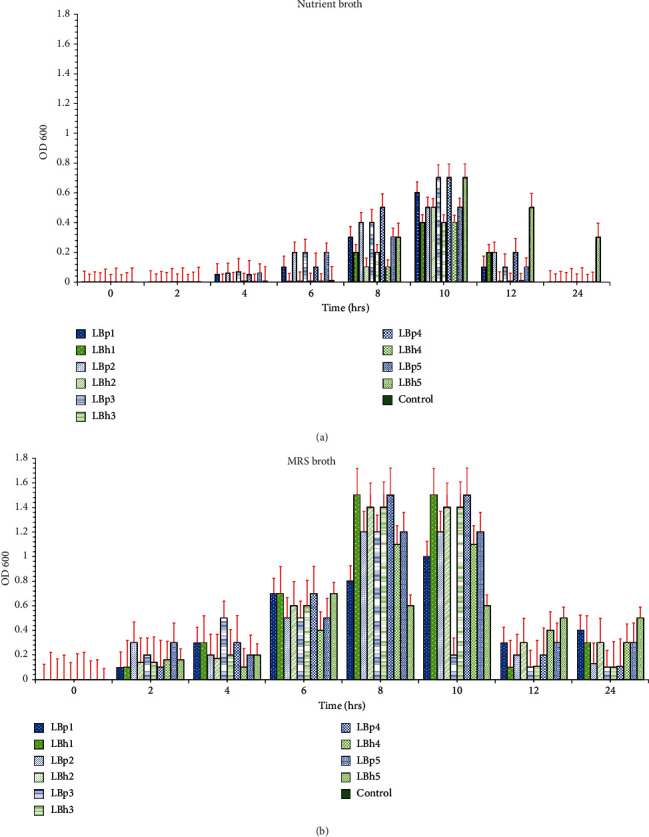
Growth curve of *L. plantarum* and *L. helveticus* isolates. (a) Nutrient broth. (b) MRS broth. LBp1-LBp5 are the *L. plantarum* isolates, and LBh1-LBh5 are the *L. helveticus* isolates.

Optical densities for isolates were measured every 2 hrs, i.e., 0, 2, 4, 6, 8, 10, 12, and 24 hrs, on MRS and nutrient broth simultaneously. After 6 hours, all the isolates used in the study reached an OD of approximately 0.7 in MRS broth, while in the nutrient broth, it took 10 hours, as shown in Figure 1. In the study of Cho et al. [37], the isolates of *L. plantarum* reached stationary phase after 10 hours. Balamurugan et al. [38] also reported that *L. helveticus* showed an elevated growth curve at around 16 hours in MRS broth, which was similar to our results as shown in Tables S1 and S2.

### 3.3. Evaluation of Probiotic Properties

#### 3.3.1. pH Tolerance

**Figure 2:**
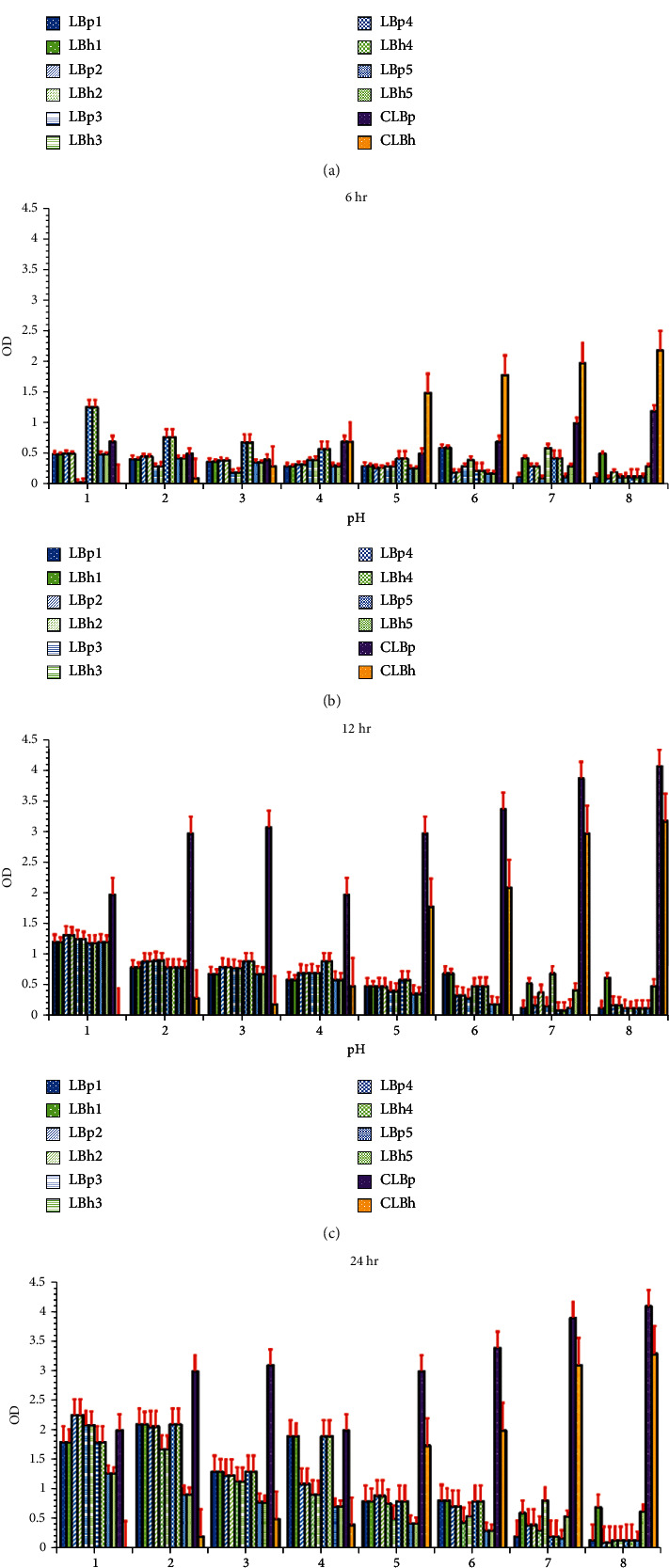
pH tolerance to *L. plantarum* and *L. helveticus* isolates: (a) 4 hours, (b) 6 hours, (c) 12 hours, and (d) 24 hours. The red bars show the standard error. LBp1-LBp5 are the *L. plantarum* isolates, LBh1-LBh5 are the *L. helveticus* isolates, CLBp was the control of *L. plantarum*, and CLBh was control of *L. helveticus.*

A low pH tolerance test is essential to predict the survival of the isolates in the stomach environment. It was observed that *L. plantarum* isolates showed negligible growth up to pH 4, but at pH 5-6, an increase in growth rate was observed. The decline in the growth rate of *L. plantarum* isolates occurred at pH above 6; the growth rate declines drastically. Chakraborty and Bhowal [39] reported that *L. plantarum* showed maximum growth at pH 5-6, which was quite similar to our findings. *Lactobacillus helveticus* isolates showed tolerance up to pH 8. Guetouache and Guessas [40] had also reported that *L. helveticus* showed tolerance at alkaline pH. Most of the isolates showed a decrease in the tolerance towards pH up to 6 hours of incubation; after that, they showed a sharp decline in their growth rate, as shown in Figure 2. *Lactobacillus helveticus* isolates showed better tolerance to high pH as compared to *L. plantarum.* The isolate LBh5 showed higher tolerance against higher pH (up to pH 8) as well as a lower pH as compared to other isolates used in this study. LBh1 showed the most significant results, among all other isolates. The statistical analysis is as given in Tables S3 and S4.

#### 3.3.2. NaCl Tolerance

**Figure 3:**
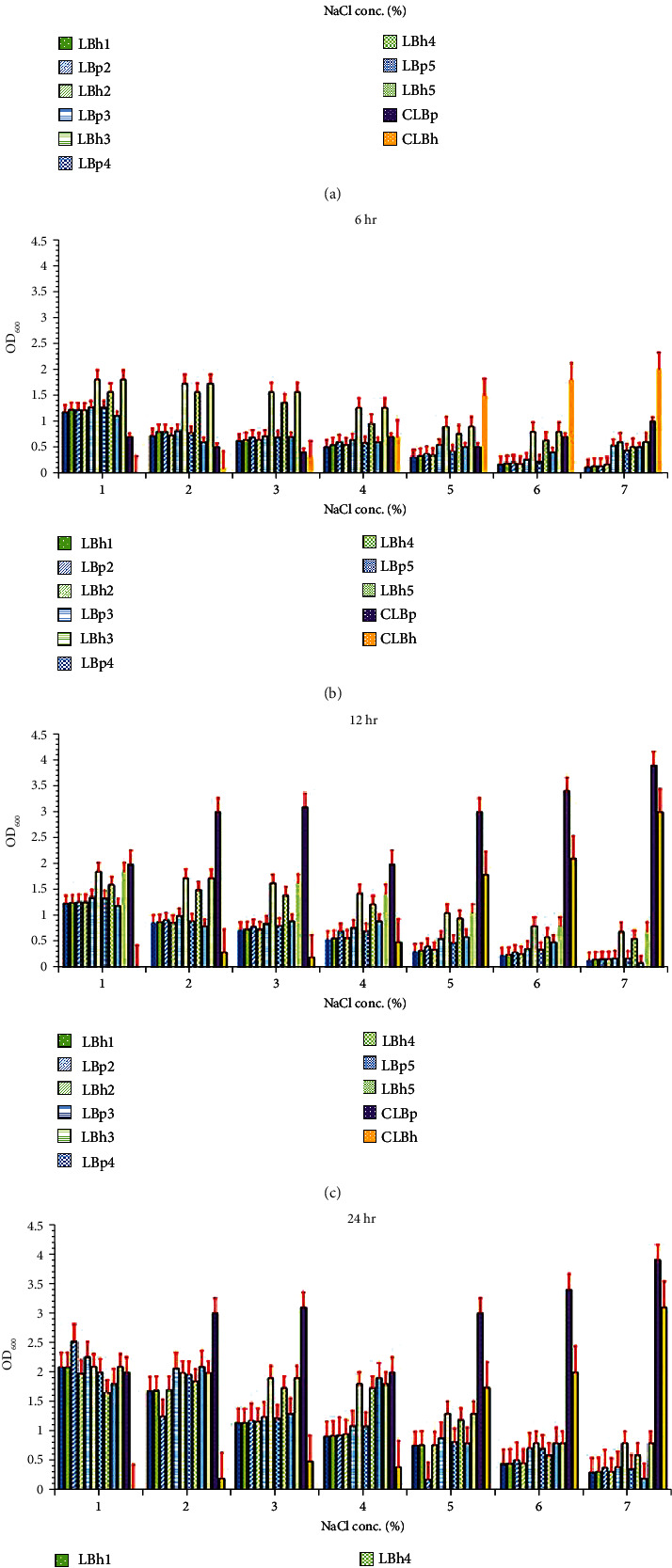
NaCl tolerance of *L. plantarum and L. helveticus* isolates: (a) 4 hours, (b) 6 hours, (c) 12 hours, and (d) 24 hours. The red bars show the standard error. LBp1-LBp5 are the *L. plantarum* isolates, LBh1-LBh5 are the *L. helveticus* isolates, CLBp was the control of *L. plantarum*, and CLBh was control of *L. helveticus.*

Tolerance to a high concentration of NaCl is necessary for a probiotic to be effective in the human gut. NaCl tolerance was considered to be an important parameter because NaCl concentration is a scale to measure how much bacteria are able to tolerate toxic and osmotic shock. For both the *Lactobacillus* spp., optical densities decrease gradually with an increase in NaCl concentrations, as shown in Figure 3. *Lactobacillus plantarum* isolates comparatively showed higher OD values than *L. helveticus* isolates. Both lactobacilli showed growth at 1-4% NaCl concentration, while at a higher concentration of NaCl, from 6 to 7%, bacterial growth was decreased. Balamurugan et al. [38] also reported that *L. helveticus* showed tolerance to NaCl concentration from 1 to 7%, and Chowdhury et al. [41] observed that *L. plantarum* was able to tolerate NaCl concentration from 1 to 9%: both of these studies matched our findings. The isolates LBh5 showed the most substantial tolerance to NaCl concentration as compared to other isolates of *L. helveticus.* In *L. plantarum* isolates, LBp3 showed the most favourable results among the others, as shown in Figure 3; it was concluded that LBh5 showed the most positive results as compared to all isolates of *L. plantarum* and *L. helveticus* used in this study. The results were statistically analysed as shown in Tables S5 and S6.

#### 3.3.3. Bile Salt Tolerance

**Figure 4:**
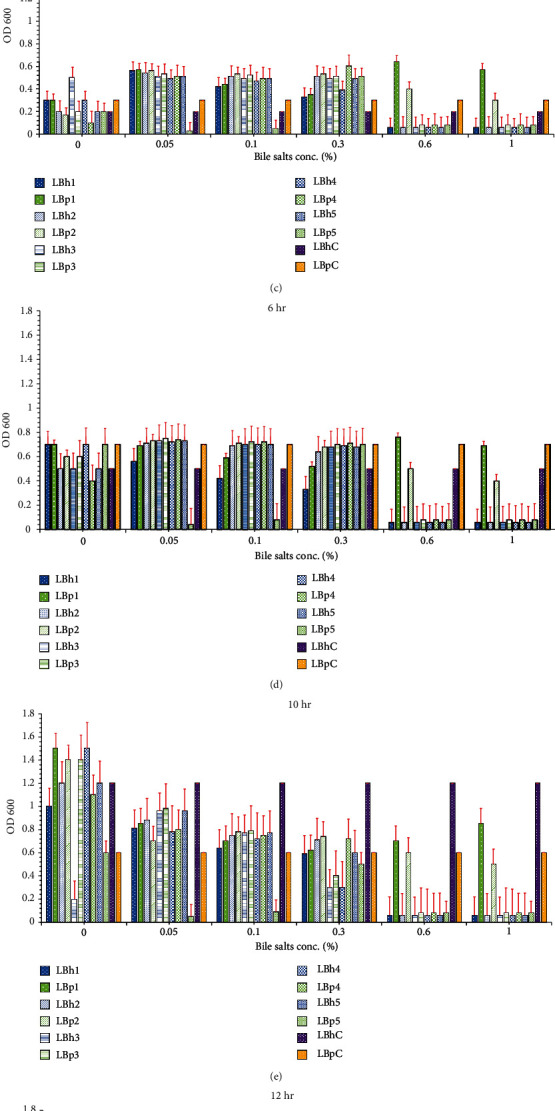
Bile salt tolerance of *L. plantarum and L. helveticus* isolates at different times: (a) 0 hours, (b) 2 hours, (c) 4 hours, (d) 6 hours, (e) 10 hr, (f) 12 hr, and (g) 24 hr. The red bars show the standard error. LBp1-LBp5 are the *L. plantarum* isolates, LBh1-LBh5 are the *L. helveticus* isolates, CLBp was the control of *L. plantarum*, and CLBh was control of *L. helveticus.*

Bile salt tolerance is an essential factor as it determines the survival of probiotics in the intestine. The bile salts present in the intestinal tract disrupt the cell membrane of bacteria entering the stomach. Probiotics have the ability to tolerate 0.05 to 0.3% of bile. The results calculated showed that the maximum growth of isolates was observed in the presence of bile salts up to 0.3%, as shown in Figure 4. The isolates, however, were less tolerant of the higher concentrations, i.e., 0.6% and 1%. Chowdhury et al. [41] observed that *Lactobacillus* subsp. isolated from yoghurt tolerated around 0.3% of bile concentrations. *Lactobacillus helveticus* isolates showed tolerance up to 0.3%, and maximum tolerance was observed at 0.1%. Barua et al. [42] and Rong et al. [43] observed that *Lactobacillus* spp. showed maximum growth at 0.1%, and maximum tolerance was shown at 0.3% concentration, which correlated to this study. Similar findings were reported in a work done by Baick and Kim [44]. The isolates LBp1 and LBp2 were able to withstand a higher bile salt concentration up to 1% as compared to other isolates of *L. helveticus* and *L. plantarum*. On the contrary, the findings of Succi et al. [45] showed the survival of lactobacillus bacteria at high bile concentrations up to 1%. The isolate LBh4 showed maximum tolerance, i.e., up to 0.3% as compared to other isolates of *L. helveticus*. It was concluded that *L. plantarum* and *L. helveticus* isolates can be considered to be suitable probiotic isolates as they tolerated 0.3% of bile concentration, which is typical of healthy men [9]. The statistical analysis of the result is shown in Tables S7 and S8.

#### 3.3.4. Resistance to Antibiotics

**Figure 5:**
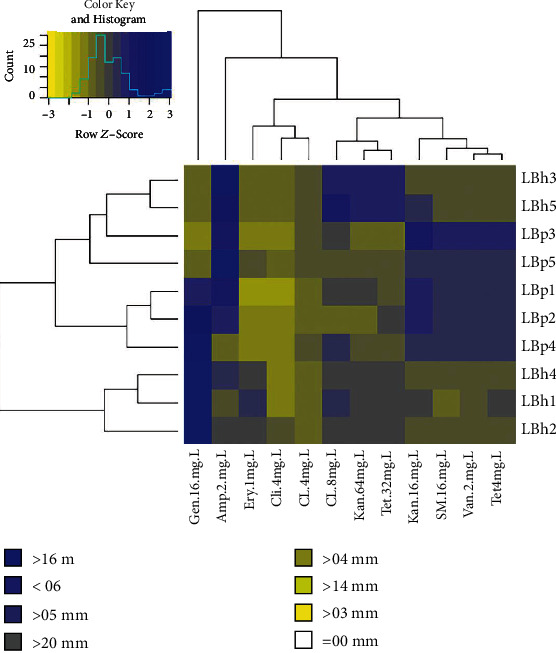
Antibiotic susceptibility of the isolates of *L. plantarum* (LBp) and *L. helveticus* (LBh): ampicillin (Amp.), vancomycin (Van), gentamicin (Gen), kanamycin, (Kan), streptomycin (SM), erythromycin (Ery), clindamycin (Cli), tetracycline (Tet), and chloramphenicol (CL). The tree is a dendrogram, which shows the hierarchy (similarity) between isolates and their activity against the different antibiotics. The graph was plotted using the R program.

Potential probiotic bacteria show a natural resistance to antibiotics. All *L. plantarum* isolates exhibited resistance towards clindamycin. Anas et al. [46] reported similar results in their study. The *L. plantarum* isolates showed sensitivity against ampicillin, gentamycin, and other antibiotics, and among all isolates, LBp1 showed the maximum zone of inhibition against kanamycin (38.1 mm), as shown in Figure S1. The isolates LBp3 and LBp5 showed resistance against gentamycin, kanamycin, erythromycin, clindamycin, and tetracycline. It was concluded that among these five isolates, LBp3 and LBp5 were resistant against most of the drugs. Similar observations were reported by Somashekaraiah et al. [47]. LBh3 and Lbh5 showed resistance against vancomycin, gentamycin, streptomycin, erythromycin, and clindamycin, as shown in Figure 5. All the isolates of *L. helveticus* showed sensitivity towards kanamycin and ampicillin. In the case of clindamycin, all the four isolates of *L. helveticus* showed resistance except LBh2 isolate, which was sensitive to this drug. The isolate LBh2 was sensitive to gentamycin with a maximum zone of inhibition of 25.7 mm. The antibiotic susceptibility tests showed that *L. helveticus* isolates had better probiotic properties as compared to *L. plantarum* isolates. Most of the *L. helveticus* isolates were found to be sensitive to all antibiotics used in this study as compared to *L. plantarum* isolates. Resistance to various antibiotics would help the isolates settle in gut microflora for longer; therefore, may alleviate gastrointestinal tract-related side effects due to antibiotics. Tables S9 and S10 show the statistical analysis of the results.

#### 3.3.5. Antibacterial Activity

**Figure 6:**
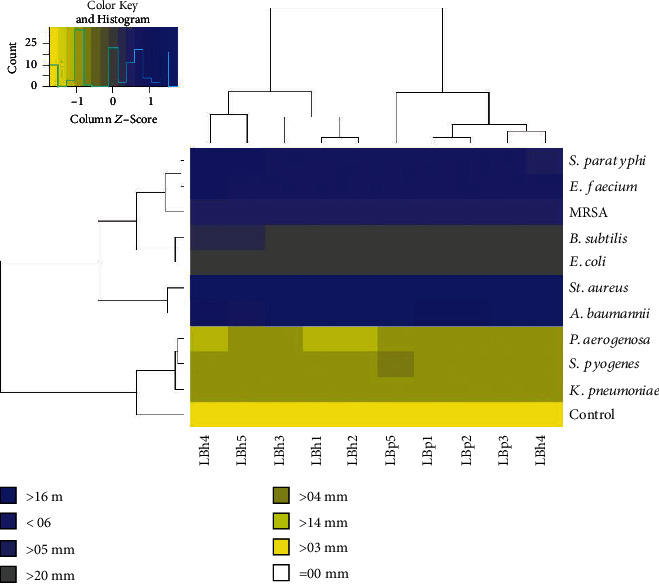
Antibacterial activity of different *L. plantarum* and *L. helveticus* isolates against foodborne pathogens. The tree is a dendrogram, which shows the hierarchy (similarity) between isolates and their activity against the different foodborne pathogens. The graph was plotted using R program.

The antibacterial activity of *L. plantarum* and *L. helveticus* was measured against foodborne pathogens. All *L. plantarum* and *L. helveticus* isolates showed a maximum zone of inhibition against *St. aureus* (22.9 mm and 19.5 mm, respectively). In contrast, minimum zones were found against the gram-positive bacterium *S. pyogenes* (6.8 mm and 4.8 mm), as shown in Figure 6 and Figure S2. Al-Madboly and Abdullah [48] reported that *L. plantarum* showed weaker zones of inhibition against *St. aureus* and stronger zones of inhibition against *E. coli*, *Bacillus cereus*, and *Salmonella typhii*, which contradicts the results of this study. Succi et al. [45] found *L. plantarum* had more potent antagonistic activity against *St. aureus*, which correlates with our studies. In the case of gram-negative bacteria, the *L. plantarum* and *L. helveticus* isolates showed maximum zones of inhibition against *A. baumannii* (22.9 mm and 19.1 mm). The *L. plantarum* isolates showed the minimum zone of inhibition against *K. pneumoniae* (5 mm), and *L. helveticus* isolates showed a minimum zone of inhibition against *P. aeruginosa* (5.6 mm). The isolates LBh5 showed significant results against gram-positive bacteria, while LBh3 isolates showed the most promising results against gram-negative bacteria as compared to other *L. helveticus* isolates. Gupta et al. [49] observed the same results in the case of *L. helveticus* antibacterial activity. The isolate LBp5 showed the most promising results against both gram-positive and gram-negative bacteria in comparison to all other isolates used in this study. Tables S11 and S12 present detailed results of the Pearson correlations.

#### 3.3.6. Response to Stomach-Duodenal Stimulus

**Figure 7:**
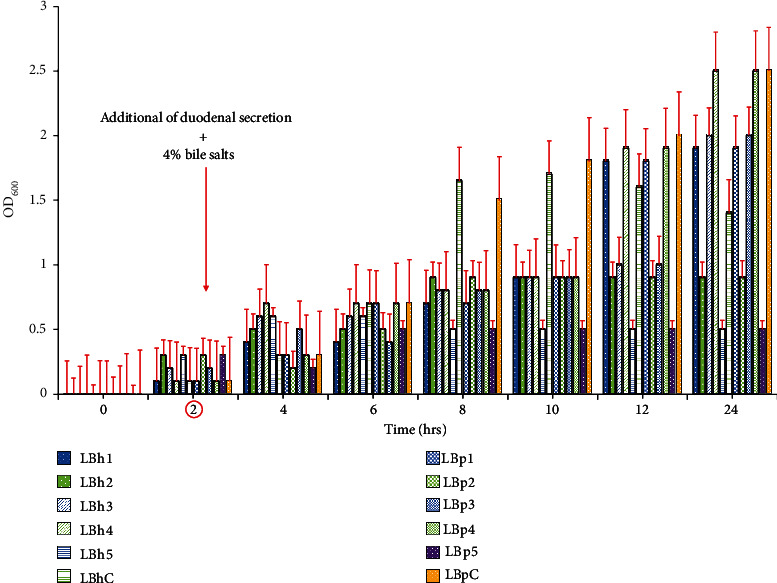
Response to stomach-duodenal stimulus of *L. plantarum* and *L. helveticus* isolates at different times. At 2 hours, duodenal secretion + 4 percent bile salts were added.

The response of lactic acid bacteria isolates to stomach-duodenal stimulus is shown in Figure 7. These results indicated that there was a resistance of most isolates to adverse conditions imposed by the composition of this medium. All the tested isolates were resistant to pH 3 after 2 hrs of incubation. Similar results were reported by Vizoso Pinto et al. [34], although their isolates showed resistance after 1 hour of incubation.

#### 3.3.7. Arginine Hydrolysis Test

Both the isolates were shown to be arginine-positive as on a white background they displayed a bright orange colour.

## 4. Conclusion

Bacteriocin-producing lactic acid bacteria were successfully isolated from traditional Pakistani yoghurt. The experimental results showed that both *L. plantarum* and *L. helveticus* isolates were able to tolerate low pH, high bile salt, and NaCl concentrations. They also showed significant antimicrobial activity against test foodborne microorganisms and were resistant to many antibiotics. Thus, our isolates proved that they bear probiotic potential and could be used in different food items as probiotics. The *L. helveticus* isolates (LBh5) showed good probiotic properties as compared to other isolates investigated in this study. A further study on these isolates is needed to explore more about their role in increasing the shelf life of food or preventing or treating any gastrointestinal infections. The data was authenticated by replication. The Pearson correlation was applied which showed most of the results with significant values.

### 4.1. Limitations of the Study

Despite the promising results and uniqueness of our study, these isolates should be further studied to prove their importance in the dairy industry. It would be valuable to test the following characteristics: (i) molecular identification of isolates; (ii) adhesion to mucosal surfaces; (iii) clinical studies for human health; and (iv) technological properties (strain stability, viability into products, and bacteriophage resistance).

## Data Availability

The authors will provide the data if needed.

## Supplementary Materials

Supplementary Materials The supplementary files consist of antibiotic susceptibility test tables of *L. plantarum* and *L. helveticus* species and their pictorial representation used in this study. It also contains the tables of growth rate curve, tolerance against NaCl, pH, bile salts, response to stomach duodenal stimulus, antibacterial activity, and antibiotic resistance of *L. plantarum* and *L. helveticu*s isolates against gram-positive and gram-negative foodborne bacteria and a pictorial representation of antibacterial activity for *L. plantarum* and *L*. *helveticus* species against *Staphylococcus aureus* species.

## References

[1] Sriphannam C., Kummasook A. (2020). Evaluation of probiotic properties of lactic acid bacteria isolated from kimchi produced in Iran. *Naresuan University Journal: Science and Technology*.

[2] Ghosh T., Beniwal A., Semwal A., Navani N. K. (2019). Mechanistic insights into probiotic properties of lactic acid bacteria associated with ethnic fermented dairy products. *Frontiers in Microbiology*.

[3] Hickey C. D., Sheehan J. J., Wilkinson M. G., Auty M. A. E. (2015). Growth and location of bacterial colonies within dairy foods using microscopy techniques: a review. *Frontiers in Microbiology*.

[4] Indira M., Venkateswarulu T. C., Vidya Prabhakar K., Abraham Peele K., Krupanidhi S. (2018). Isolation and characterization of bacteriocin producing *Enterococcus casseliflavus* and its antagonistic effect on *Pseudomonas aeruginosa*. *Karbala International Journal of Modern Science*.

[5] Motevaseli E., Dianatpour A., Ghafouri-Fard S. (2017). The role of probiotics in cancer treatment: emphasis on their *in vivo* and *in vitro* anti-metastatic effects. *International Journal of Molecular and Cellular Medicine*.

[6] Delgado S., Sánchez B., Margolles A., Ruas-Madiedo P., Ruiz L. (2020). Molecules produced by probiotics and intestinal microorganisms with immunomodulatory activity. *Nutrients*.

[7] Rossi M., Amaretti A., Raimondi S. (2011). Folate production by probiotic bacteria. *Nutrients*.

[8] Issazadeh K., Ali Abadi M. A., Kazemi Darsanaki R., Alikhani F., Dadras H., Tajehmiri A. (2013). Isolation, identification and analysis of probiotic properties of *Lactobacillus* spp. from traditional yoghurts in north of Iran. *Journal of Pure and Applied Microbiology*.

[9] Goel A., Halami P. M., Tamang J. P. (2020). Genome analysis of *Lactobacillus plantarum* isolated from some Indian fermented foods for bacteriocin production and probiotic marker genes. *Frontiers in Microbiology*.

[10] Papadimitriou K., Zoumpopoulou G., Foligné B. (2015). Discovering probiotic microorganisms: in vitro, in vivo, genetic and omics approaches. *Frontiers in Microbiology*.

[11] Rehaiem A., Belgacem Z. B., Edalatian M. R. (2014). Assessment of potential probiotic properties and multiple bacteriocin encoding-genes of the technological performing strain *Enterococcus faecium* MMRA. *Food Control*.

[12] Zhou T., Huo R., Kwok L.-Y. (2019). Effects of applying *Lactobacillus helveticus* H9 as adjunct starter culture in yogurt fermentation and storage. *Journal of Dairy Science*.

[13] Li C., Song J., Kwok L. Y. (2017). Influence of *Lactobacillus plantarum* on yogurt fermentation properties and subsequent changes during postfermentation storage. *Journal of Dairy Science*.

[14] Kaboosi H. (2011). Antibacterial effects of probiotics isolated from yoghurts against some common bacterial pathogens. *African Journal of Microbiology Research*.

[15] Gerbaldo G. A., Barberis C., Pascual L., Dalcero A., Barberis L. (2012). Antifungal activity of two *Lactobacillus* strains with potential probiotic properties. *FEMS Microbiology Letters*.

[16] Reuben R. C., Roy P. C., Sarkar S. L., Rubayet Ul Alam A. S. M., Jahid I. K. (2020). Characterization and evaluation of lactic acid bacteria from indigenous raw milk for potential probiotic properties. *Journal of Dairy Science*.

[17] Rubbani U., Iqbal A. (2020). Evaluation of isolated *Lactobacillus* strains as probiotics in yogurt preparation. *Advancements in Life Sciences*.

[18] Aslam S., Qazi J. I. (2010). Isolation of acidophilic lactic acid bacteria antagonistic to microbial contaminants. *Pakistan Journal of Zoology*.

[19] Afzaal S., Hameed U., Ahmad N., Rashid N., Haider M. S. (2019). Molecular identification and characterization of lactic acid producing bacterial strains isolated from raw and traditionally processed foods of Punjab, Pakistan. *Pakistan Journal of Zoology*.

[20] Akram M. F. S., Ashraf M., Ali S., Kazmi S. I. (2017). Isolation of gram-positive bacteria from different sources and evaluation of their probiotic properties. *Journal of Medical Microbiology and Infectious Diseases*.

[21] Ashraf M., Arshad M., Siddique M., Muhammad G. (2009). In vitro screening of locally isolated *Lactobacillus* species for probiotic properties. *Pakistan Veterinary Journal*.

[22] Zahid M., Ashraf M., Arshad M., Muhammad G., Yasmin A., Hameed H. M. A. (2015). Antimicrobial activity of bacteriocins isolated from lactic acid bacteria against resistant pathogenic strains. *International Journal of Nutrition and Food Sciences*.

[23] Goraya M. U., Ashraf M., Ur Rahman S., Raza A., Habib A. (2013). Determination of antibacterial activity of bacteriocins of lactic acid producing bacteria. *Journal of Infection and Molecular Biology*.

[24] Aslam M., Shahid M., Rehman F. U. (2011). Purification and characterization of bacteriocin isolated from *Streptococcus thermophilus*. *African Journal of Microbiology Research*.

[25] Tufail M., Hussain S., Malik F. (2011). Isolation and evaluation of antibacterial activity of bacteriocin produced by *Lactobacillus bulgaricus* from yogurt. *African Journal of Microbiology Research*.

[26] Ali J., Hussain A., Rehman S. (2018). Isolation, identification and characterization of lactic acid bacteria from traditional yogurt. *Specialty Journal of Biological Sciences*.

[27] Ibrahim F., Aman A., Siddiqui N. N., Zohra R. R., Ul Qader S. A., Ansari A. (2019). Screening of antilisterial efficacy and partial purification of chromosomally located bacteriocin isolated from *Lactobacillus plantarum*. *International Journal of Biology and Biotechnology*.

[28] Aziz G., Zaidi A., Bakht U. (2020). Microbial safety and probiotic potential of packaged yogurt products in Pakistan. *Journal of Food Safety*.

[29] Hassan M. U., Nayab H., Rehman T. U. (2020). Characterisation of bacteriocins produced by *Lactobacillus* spp. isolated from the traditional Pakistani yoghurt and their antimicrobial activity against common foodborne pathogens. *BioMed Research International*.

[30] Yang Y., Greenleaf Z., Kann W., Sha M. (2019). Microaerobic fermentation of *Lactobacillus acidophilus* within gut microbiome physiological conditions by BioFlo ® Bioprocess control stations. *Application Notes – Eppendorf*.

[31] Leong Y., Ker P., Jamaludin M. (2018). UV-Vis spectroscopy: a new approach for assessing the color index of transformer insulating oil. *Sensors*.

[32] Jorgensen J. H., Hindler J. F., Reller L. B., Weinstein M. P. (2007). New consensus guidelines from the clinical and laboratory standards institute for antimicrobial susceptibility testing of infrequently isolated or fastidious bacteria. *Clinical Infectious Diseases*.

[33] Rychen G., Aquilina G., Azimonti G. (2018). Guidance on the characterisation of microorganisms used as feed additives or as production organisms. *EFSA Journal*.

[34] Vizoso Pinto M. G., Franz C. M. A. P., Schillinger U., Holzapfel W. H. (2006). *Lactobacillus* spp. with in vitro probiotic properties from human faeces and traditional fermented products. *International Journal of Food Microbiology*.

[35] N'tcha C., Haziz S., Agbobatinkpo P. (2016). Probiotic properties of lactic acid bacteria isolated from a Beninese traditional beer's ferment. *International Journal of Applied Biology and Pharmaceutical Technology*.

[36] Shaik M., Shah G. (2007). Determination of probiotic properties of lactic acid bacteria from human. *Global Journal of Biology, Agriculture & Health Sciences, October*.

[37] Cho Y. H., Shin I. S., Hong S. M., Kim C. H. (2015). Production of functional high-protein beverage fermented with lactic acid bacteria isolated from Korean traditional fermented food. *Korean Journal for Food Science of Animal Resources*.

[38] Balamurugan R., Chandragunasekaran A. S., Chellappan G., Rajaram K., Ramamoorthi G., Ramakrishna B. S. (2014). Probiotic potential of lactic acid bacteria present in home made curd in southern India. *Indian Journal of Medical Research*.

[39] Chakraborty A., Bhowal J. (2015). Isolation, identification and analysis of probiotic properties of *Lactobacillus* spp. from selected regional dairy products. *International Journal of Current Microbiology and Applied Sciences*.

[40] Guetouache M., Guessas B. (2015). Characterization and identification of lactic acid bacteria isolated from traditional cheese (Klila) prepared from cows milk. *African Journal of Microbiology Research*.

[41] Chowdhury A., Hossain M. N., Mostazir N. J., Fakruddin M., Billah M. M., Ahmed M. M. (2012). Screening of *Lactobacillus* spp. from buffalo yoghurt for probiotic and antibacterial activity. *Journal of Bacteriology & Parasitology*.

[42] Barua R., Al Masud H. M. A., Mahmud N., Hakim M. A. (2015). Assessment of potential probiotic *Lactobacillus* strains isolated from goat milk. *Journal of Pharmaceutical and Biological Sciences*.

[43] Rong J., Zheng H., Liu M. (2015). Probiotic and anti-inflammatory attributes of an isolate *Lactobacillus helveticus* NS8 from Mongolian fermented koumiss. *BMC Microbiology*.

[44] Baick S. C., Kim C.-H. (2015). Assessment of characteristics and functional properties of *Lactobacillus* species isolated from kimchi for dairy use. *Korean Journal for Food Science of Animal Resources*.

[45] Succi M., Tremonte P., Reale A. (2005). Bile salt and acid tolerance of *Lactobacillus rhamnosus strains* isolated from Parmigiano Reggiano cheese. *FEMS Microbiology Letters*.

[46] Anas M., Ahmed K., Mebrouk K. (2014). Study of the antimicrobial and probiotic effect of *Lactobacillus plantarum* (P6) isolated from raw goat's milk from the region of western Algeria. *World Applied Sciences Journal*.

[47] Somashekaraiah R., Shruthi B., Deepthi B. V., Sreenivasa M. Y. (2019). Probiotic properties of lactic acid bacteria isolated from neera: a naturally fermenting coconut palm nectar. *Frontiers in Microbiology*.

[48] Al-Madboly L. A., Abdullah A. K. (2015). Potent antagonistic activity of Egyptian *Lactobacillus plantarum* against multiresistant and virulent food-associated pathogens. *Frontiers in Microbiology*.

[49] Gupta S., Awasthi S., Kumar S., Bhatnagar T. (2015). A study on probiotic potential of lactic acid bacteria (LAB) and evaluating their effectiveness against enteric and fungal infections. *International Journal Of Advanced Multidisciplinary Research*.

