# Supplementary material for: Probiotic Properties of Lactobacillus helveticus and Lactobacillus plantarum Isolated from Traditional Pakistani Yoghurt
Source: Biomed Res Int. 2020 Dec 24;2020:8889198. doi: 10.1155/2020/8889198 (PMC7775145; doi:10.1155/2020/8889198)
Supplement: Supplementary Materials — The supplementary files consist of antibiotic susceptibility test tables of L. plantarum and L. helveticus species and their pictorial representation used in this study. It also contains the tables of growth rate curve, tolerance against NaCl, pH, bile salts, response to stomach duodenal stimulus, antibacterial activity, and antibiotic resistance of L. plantarum and L. helveticus isolates against gram-positive and gram-negative foodborne bacteria and a pictorial representation of antibacterial activity for L. plantarum and L. helveticus species against Staphylococcus aureus species. [file 8889198.f1.docx]

**Supplementary tables and figures**

**Table S1: Growth rate study of the isolates of *L. helveticus* and *L. plantarum* strains in MRS broth*.***

| **Isolates** | **Time (hrs)** | | | | | | | | **Standard Deviation**  **SD** | **Pearson Correlation b/w Dose and Activity (P=0.05)** | **R squared**  **(r^2^)** |
| --- | --- | --- | --- | --- | --- | --- | --- | --- | --- | --- | --- |
|  | **0** | **2** | **4** | **6** | **8** | **10** | **12** | **24** |  |  |  |
| **LBh1** | 0.0009 | 0.1 | 0.3 | 0.7 | 1.65 | 1.7 | 1.6 | 1.4 | 0.70 | 0.017547 | 0.7917 |
| **LBh2** | 0.0001 | 0.14 | 0.17 | 0.96 | 1.4 | 1.6 | 1.8 | 1.5 | 0.73 | 0.007342 | 0.8634 |
| **LBh3** | 0.0001 | 0.14 | 0.2 | 1 | 1.4 | 1.6 | 1 | 1 | 0.66 | 0.021384 | 0.7711 |
| **LBh4** | 0.0009 | 0.16 | 0.1 | 0.4 | 1.3 | 1.5 | 1 | 1.6 | 0.66 | .015221 | 0.8055 |
| **LBh5** | 0.0001 | 0.16 | 0.2 | 0.3 | 0.5 | 1.2 | 1.9 | 2.9 | 1.03 | 0.011124 | 0.8328 |
| **LBp1** | 0.0009 | 0.1 | 0.3 | 0.7 | 1.5 | 1.8 | 2 | 2.5 | 0.95 | 0.000441 | 0.9659 |
| **LBp2** | 0.0001 | 0.3 | 0.2 | 0.5 | 1.8 | 2.11 | 3 | 3.5 | 1.36 | 0.000441 | 0.9251 |
| **LBp3** | 0.0001 | 0.2 | 0.5 | 0.4 | 1.74 | 2 | 3.1 | 3.6 | 1.38 | 0.002392 | 0.9212 |
| **LBp4** | 0.0009 | 0.1 | 0.3 | 0.7 | 1.5 | 1.8 | 2 | 2.5 | 0.95 | 0.000441 | 0.9659 |
| **LBp5** | 0.0001 | 0.3 | 0.2 | 0.5 | 1.8 | 2.11 | 3 | 3.5 | 1.36 | 0.002252 | 0.9251 |
| **Control** | 0 | 0 | 0 | 0 | 0 | 0 | 0 | 0 | 0 | 0 | 0 |

**Table S2: Growth rate study of the isolates of *L. helveticus* and *L. plantarum* strains in nutrient broth*.***

| **Isolates** | **Time (hours)** | | | | | | | | **Standard Deviation**  **SD** | **Pearson Correlation b/w Dose and Activity (P=0.05)** | **R squared**  **(r^2^)** |
| --- | --- | --- | --- | --- | --- | --- | --- | --- | --- | --- | --- |
|  | **0** | **2** | **4** | **6** | **8** | **10** | **12** | **14** |  |  |  |
| **LBh1** | 0.0001 | 0.001 | 0.002 | 0.004 | 0.008 | 0.2 | 0.4 | 0.5 | 0.20 | 0.025768 | 0.7498 |
| **LBh2** | 0.0001 | 0.002 | 0.003 | 0.006 | 0.008 | 0.1 | 0.5 | 0.8 | 0.30 | 0.054338  Not significant | 0.645 |
| **LBh3** | 0.0001 | 0.003 | 0.006 | 0.007 | 0.008 | 0.2 | 0.4 | 0.5 | 0.20 | 0.025731 | 0.75 |
| **LBh4** | 0.0001 | 0.003 | 0.004 | 0.007 | 0.009 | 0.1 | 0.2 | 0.5 | 0.17 | 0.059561  Not significant | 0.75 |
| **LBh5** | 0.0001 | 0.003 | 0.007 | 0.008 | 0.008 | 0.3 | 0.5 | 0.8 | 0.30 | 0.028696 | 0.7367 |
| **LBp1** | 0.0001 | 0.002 | 0.05 | 0.1 | 0.4 | 0.5 | 2.2 | 2.59 | 1.04 | 0.029892 | 0.7315 |
| **LBp2** | 0.0001 | 0.003 | 0.06 | 0.2 | 0.3 | 0.8 | 2.3 | 2.7 | 1.08 | 0.022041 | 0.7677 |
| **LBp3** | 0.0001 | 0.003 | 0.07 | 0.3 | 0.2 | 0.9 | 2.5 | 2.8 | 1.15 | 0.023491 | 0.7606 |
| **LBp4** | 0.0001 | 0.002 | 0.05 | 0.1 | 0.4 | 0.5 | 2.2 | 2.59 | 1.04 | 0.029892 | 0.7315 |
| **LBp5** | 0.0001 | 0.003 | 0.06 | 0.2 | 0.3 | 0.8 | 2.3 | 2.7 | 1.08 | 0.022041 | 0.7677 |
| **Control** | 0 | 0 | 0 | 0 | 0 | 0 | 0 | 0 | 0 | 0 | 0 |

**Table S3: pH tolerance of the isolates of *L. helveticus.***

| **Isolates** | **pH** | **Time (hr)** | | | | **Standard deviation SD** | **Pearson Correlation b/w Dose and Activity (P=0.05)** | **R squared**  **(r^2^)** |
| --- | --- | --- | --- | --- | --- | --- | --- | --- |
|  |  | **4** | **6** | **12** | **24** |  |  |  |
| **LBh1** | 1 | 0.03 | 0.49 | 1.216 | 1.8 | 0.78 | 0.001616 | 0.9351 |
|  | 2 | 0.03 | 0.41 | 0.8 | 1.8 | 0.76 | 0.000264 | 0.9736 |
|  | 3 | 0.03 | 0.37 | 0.69 | 1.3 | 0.54 | 0.000323 | 0.9708 |
|  | 4 | 0.035 | 0.29 | 0.6 | 1.9 | 0.82 | 0.00062 | 0.9596 |
|  | 5 | 0.21 | 0.3 | 0.5 | 0.8 | 0.26 | 0.00028 | 0.9728 |
|  | 6 | 0.023 | 0.6 | 0.7 | 0.81 | 0.35 | 0.043066 | 0.6813 |
|  | 7 | 0.3 | 0.43 | 0.54 | 0.6 | 0.13 | 0.037374 | 0.7017 |
|  | 8 | 0.3 | 0.5 | 0.63 | 0.7 | 0.17 | 0.032438 | 0.721 |
| **LBh2** | 1 | 0.02 | 0.50 | 1.34 | 2.26 | 0.98 | 0.0006 | 0.961 |
|  | 2 | 0.04 | 0.45 | 0.9 | 2.07 | 0.87 | 0.0002 | 0.9799 |
|  | 3 | 0.048 | 0.39 | 0.81 | 1.24 | 0.51 | 0.0012 | 0.9444 |
|  | 4 | 0.02 | 0.33 | 0.71 | 1.09 | 0.46 | .001363 | 0.9403 |
|  | 5 | 0.62 | 0.27 | 0.49 | 0.89 | 0.26 | 0.0426 | 0.6829 |
|  | 6 | 0.84 | 0.20 | 0.35 | 0.72 | 0.30 | 0.3081  Non-significant | 0.2539 |
|  | 7 | 0.3 | 0.30 | 0.39 | 0.40 | 0.05 | 0.0927  Non-significant | 0.5475 |
|  | 8 | 0.2 | 0.20 | 0.19 | 0.10 | 0.04 | 0.8487  Non-significant | 0.0102 |
| **LBh3** | 1 | 0.03 | 0.03 | 1.27 | 2.09 | 1.00 | 0.8487  Non-significant | 0.9224 |
|  | 2 | 0.028 | 0.3 | 0.92 | 1.68 | 0.73 | 0.0003 | 0.9722 |
|  | 3 | 0.05 | 0.2 | 0.79 | 1.14 | 0.50 | 0.0017 | 0.9333 |
|  | 4 | 0.035 | 0.4 | 0.71 | 0.92 | 0.38 | 0.0066 | 0.8703 |
|  | 5 | 0.61 | 0.3 | 0.42 | 0.5 | 0.13 | 0.3027  Non-significant | 0.2589 |
|  | 6 | 0.71 | 0.4 | 0.5 | 0.55 | 0.12 | 0.3594  Non-significant | 0.211 |
|  | 7 | 0.5 | 0.6 | 0.7 | 0.81 | 0.13 | 0 .053541  Non-significant | 0.6474 |
|  | 8 | 0.19 | 0.12 | 0.13 | 0.14 | 0.03 | 0.4248  Non-significant | 0.1646 |
| **LBh4** | 1 | 0.04 | 1.263 | 1.2 | 1.8 | 0.74 | 0.0282 | 0.7387 |
|  | 2 | 0.038 | 0.78 | 0.8 | 2.1 | 0.85 | 0.0019 | 0.9289 |
|  | 3 | 0.06 | 0.69 | 0.9 | 1.3 | 0.51 | 0.0081 | 0.8564 |
|  | 4 | 0.06 | 0.58 | 0.9 | 1.9 | 0.77 | 0.0003 | 0.971 |
|  | 5 | 0.6 | 0.42 | 0.6 | 0.8 | 0.155 | 0.0547  Non-significant | 0.644 |
|  | 6 | 0.69 | 0.22 | 0.5 | 0.8 | 0.25 | 0.1036  Non-significant | 0.5245 |
|  | 7 | 0.32 | 0.43 | 0.1 | 0.2 | 0.14 | 0.9531  Non-significant | 0.001 |
|  | 8 | 0.19 | 0.12 | 0.13 | 0.14 | 0.03 | 0.9531  Non-significant | 0.1646 |
| **LBh5** | 1 | 0.05 | 0.49 | 1.216 | 1.27 | 0.59 | 0.0175 | 0.7917 |
|  | 2 | 0.04 | 0.43 | 0.8 | 0.92 | 0.39 | 0.0132 | 0.8183 |
|  | 3 | 0.07 | 0.36 | 0.69 | 0.79 | 0.32 | .011225 | 0.8321 |
|  | 4 | 0.08 | 0.3 | 0.6 | 0.71 | 0.28 | 0.0076 | 0.861 |
|  | 5 | 0.5 | 0.26 | 0.372 | 0.42 | 0.10 | 0.2818  Non-significant | 0.2786 |
|  | 6 | 0.6 | 0.182 | 0.2 | 0.3 | 0.19 | 0.7656  Non-significant | 0.0248 |
|  | 7 | 0.7 | 0.3 | 0.43 | 0.54 | 0.16 | 0.353  Non-significant | 0.216. |
|  | 8 | 0.19 | 0.3 | 0.5 | 0.63 | 0.19 | 0.0047 | 0.89 |
| **Control** | 1 | 0.3 | 0.7 | 1.5 | 1.8 | 0.69 | .005275 | 0.8838 |
|  | 2 | 0.3 | 0.7 | 1.5 | 1.8 | 0.69 | .005276 | 0.8839 |
|  | 3 | 0.3 | 0.7 | 1.5 | 1.8 | 0.69 | .005277 | 0.8840 |
|  | 4 | 0.3 | 0.7 | 1.5 | 1.8 | 0.69 | .005278 | 0.8841 |
|  | 5 | 0.3 | 0.7 | 1.5 | 1.8 | 0.69 | .005279 | 0.8842 |
|  | 6 | 0.3 | 0.7 | 1.5 | 1.8 | 0.69 | .005280 | 0.8843 |
|  | 7 | 0.3 | 0.7 | 1.5 | 1.8 | 0.69 | .005281 | 0.8844 |
|  | 8 | 0.3 | 0.7 | 1.5 | 1.8 | 0.69 | .005282 | 0.8845 |

**Table S4: pH tolerance of the isolates of *L. plantarum.***

| **Isolates** | **pH** | **Time (hr)** | | | | **Standard deviation**  **SD** | **Pearson Correlation b/w Dose and Activity**  **(P=0.05)** | **R squared**  **(r^2^)** |
| --- | --- | --- | --- | --- | --- | --- | --- | --- |
|  |  | **4** | **6** | **12** | **24** |  |  |  |
|  | 1 | 0.03 | 0.49 | 1.216 | 1.8 | 0.78 | .014844 | 0.9351 |
| **LBp1** | 2 | 0.03 | 0.41 | 0.8 | 2.1 | 0.90 | 0.000264 | 0.9736 |
|  | 3 | 0.03 | 0.37 | 0.69 | 1.3 | 0.54 | 0.000323 | 0.9708 |
|  | 4 | 0.035 | 0.29 | 0.6 | 1.9 | 0.26 | 0.00062 | 0.9596 |
|  | 5 | 0.21 | 0.3 | 0.5 | 0.8 | 0.26 | 0.00028 | 0.9728 |
|  | 6 | 0.023 | 0.6 | 0.7 | 0.7 | 0.32 | 0.043066 | 0.6813 |
|  | 7 | 0.28 | 0.123 | 0.14 | 0.2 | 0.07 | 0.445677  Non-significant | 0.1515 |
|  | 8 | 0.19 | 0.12 | 0.13 | 0.14 | 0.03 | 0.424838  Non-significant | 0.1646 |
| **LBp2** | 1 | 0.02 | 0.50 | 1.34 | 2.26 | 0.98 | 0.000578 | 0.961 |
|  | 2 | 0.04 | 0.45 | 0.9 | 2.07 | 0.87 | 0.000152 | 0.9799 |
|  | 3 | 0.048 | 0.39 | 0.81 | 1.24 | 0.51 | 0.001182 | 0.9444 |
|  | 4 | 0.02 | 0.33 | 0.71 | 1.09 | 0.46 | 0.001363 | 0.9403 |
|  | 5 | 0.62 | 0.27 | 0.49 | 0.89 | 0.26 | 0.04259 | 0.6829 |
|  | 6 | 0.84 | 0.20 | 0.35 | 0.72 | 0.30 | 0.308124  Non-significant | 0.2539 |
|  | 7 | 0.7 | 0.30 | 0.179 | 0.40 | 0.22 | 0.723493  Non-significant | 0.0348 |
|  | 8 | 0.2 | 0.10 | 0.19 | 0.10 | 0.05 | 0.683185  Non-significant | 0.046 |
| **LBp3** | 1 | 0.03 | 0.03 | 1.27 | 2.09 | 1.00 | 0.0023 | 0.9224 |
|  | 2 | 0.028 | 0.3 | 0.92 | 1.68 | 0.73 | 0.0003 | 0.9722 |
|  | 3 | 0.05 | 0.2 | 0.79 | 1.14 | 0.50 | 0.0017 | 0.9333 |
|  | 4 | 0.035 | 0.4 | 0.71 | 0.92 | 0.38 | 0.0066 | 0.8703 |
|  | 5 | 0.61 | 0.3 | 0.42 | 0.76 | 0.20 | 0.0803  Non-significant | 0.5756 |
|  | 6 | 0.71 | 0.3 | 0.3 | 0.45 | 0.19 | 0.5702  Non-significant | 0.0871 |
|  | 7 | 0.31 | 0.1 | 0.17 | 0.31 | 0.10 | 0.1872  Non-significant | 0.387 |
|  | 8 | 0.19 | 0.12 | 0.13 | 0.14 | 0.03 | 0.4248  Non-significant | 0.1646 |
| **LBp4** | 1 | 0.04 | 1.263 | 1.2 | 1.8 | 0.74 | 0.0282 | 0.7387 |
|  | 2 | 0.038 | 0.78 | 0.8 | 2.1 | 0.85 | 0.0019 | 0.9289 |
|  | 3 | 0.06 | 0.69 | 0.9 | 1.3 | 0.51 | 0.0081 | 0.8564 |
|  | 4 | 0.06 | 0.58 | 0.9 | 1.9 | 0.77 | 0.0003 | 0.971 |
|  | 5 | 0.6 | 0.42 | 0.6 | 0.8 | 0.15 | 0.0547  Non-significant | 0.644 |
|  | 6 | 0.69 | 0.22 | 0.5 | 0.8 | 0.25 | 0.1036  Non-significant | 0.5245 |
|  | 7 | 0.32 | 0.43 | 0.1 | 0.2 | 0.14 | 0.9531  Non-significant | 0.001 |
|  | 8 | 0.19 | 0.12 | 0.13 | 0.14 | 0.03 | 0.4248  Non-significant | 0.1646 |
| **LBp5** | 1 | 0.05 | 0.49 | 1.216 | 1.27 | 0.59 | 0.0175 | 0.7917 |
|  | 2 | 0.04 | 0.43 | 0.8 | 0.92 | 0.39 | 0.0132 | 0.8183 |
|  | 3 | 0.07 | 0.36 | 0.69 | 0.79 | 0.32 | 0.0112 | 0.8321 |
|  | 4 | 0.08 | 0.3 | 0.6 | 0.71 | 0.28 | 0.0076 | 0.861 |
|  | 5 | 0.5 | 0.26 | 0.372 | 0.42 | 0.10 | 0.2818  Non-significant | 0.2786 |
|  | 6 | 0.6 | 0.182 | 0.2 | 0.3 | 0.19 | 0.7656  Non-significant | 0.0248 |
|  | 7 | 0.7 | 0.12 | 0.14 | 0.17 | 0.27 | 0.8371  Non-significant | 0.012 |
|  | 8 | 0.19 | 0.12 | 0.13 | 0.14 | 0.03 | 0.4248  Non-significant | 0.1646 |
| **Control** | 1 | 0.3 | 0.7 | 1.5 | 1.8 | 0.69 | 0.0053 | 0.8838 |
|  | 2 | 0.3 | 0.7 | 1.5 | 1.8 | 0.69 | 0.0053 | 0.8838 |
|  | 3 | 0.3 | 0.7 | 1.5 | 1.8 | 0.69 | 0.0053 | 0.8838 |
|  | 4 | 0.3 | 0.7 | 1.5 | 1.8 | 0.69 | 0.0053 | 0.8838 |
|  | 5 | 0.3 | 0.7 | 1.5 | 1.8 | 0.69 | 0.0053 | 0.8838 |
|  | 6 | 0.3 | 0.7 | 1.5 | 1.8 | 0.69 | 0.0053 | 0.8838 |
|  | 7 | 0.3 | 0.7 | 1.5 | 1.8 | 0.69 | 0.0053 | 0.8838 |
|  | 8 | 0.3 | 0.7 | 1.5 | 1.8 | 0.69 | 0.0053 | 0.8838 |

**Table S5: NaCl tolerance of *L. plantarum* strains**

| **Isolates** | **Time**  **(hrs)** | **NaCl conc. (%)** | | | | | | | **Standard Deviation**  **SD** | **Pearson Correlation b/w Dose and Activity**  **(P=0.05)** | **R squared**  **(r^2^)** |
| --- | --- | --- | --- | --- | --- | --- | --- | --- | --- | --- | --- |
|  |  | **1** | **2** | **3** | **4** | **5** | **6** | **7** |  |  |  |
| **LBp1** | **4** | 0.45 | 0.41 | 0.33 | 0.3 | 0.2 | 0.15 | 0.113 | 1.32 | 0.000073 | 0.000073 |
|  | **6** | 1.173 | 0.72 | 0.62 | 0.5 | 0.31 | 0.175 | 0.133 | 1.96 | 0.002252 | 0.9239 |
|  | **12** | 1.25 | 0.87 | 0.73 | 0.56 | 0.32 | 0.243 | 0.15 | 4.05 | .000787 | 0.9551 |
|  | **24** | 2.09 | 1.68 | 1.14 | 0.92 | 0.76 | 0.45 | 0.31 | 8.13 | 0.000721 | 0.9577 |
| **LBp2** | **4** | 0.49 | 0.43 | 0.36 | 0.3 | 0.26 | 0.182 | 0.12 | 1.31 | 0.00015 | 0.9968 |
|  | **6** | 1.216 | 0.8 | 0.69 | 0.6 | 0.372 | 0.2 | 0.14 | 1.94 | 0.001005 | 0.9504 |
|  | **12** | 1.27 | 0.92 | 0.79 | 0.71 | 0.42 | 0.3 | 0.17 | 4.02 | 0.000336 | 0.9716 |
|  | **24** | 2.52 | 1.25 | 1.182 | 0.94 | 0.813 | 0.52 | 0.393 | 8.12 | 0.015367 | 0.8053 |
| **LBp3** | **4** | 0.50 | 0.45 | 0.39 | 0.33 | 0.27 | 0.19 | 0.13 | 1.30 | < .00001 | 0.9966 |
|  | **6** | 1.27 | 0.82 | 0.71 | 0.64 | 0.54 | 0.26 | 0.53 | 1.90 | 0.026486 | 0.7474 |
|  | **12** | 1.36 | 1.00 | 0.85 | 0.77 | 0.56 | 0.37 | 0.18 | 4.00 | 0.000215 | 0.9773 |
|  | **24** | 2.26 | 2.07 | 1.24 | 1.09 | 0.89 | 0.72 | 0.40 | 8.07 | 0.002368 | 0.9332 |
| **LBp4** | **4** | 0.49 | 0.41 | 0.37 | 0.29 | 0.21 | 0.18 | 0.123 | 1.31 | 0.000054 | 0.9892 |
|  | **6** | 1.263 | 0.78 | 0.69 | 0.58 | 0.42 | 0.22 | 0.43 | 1.92 | 0.017485 | 0.7928 |
|  | **12** | 1.34 | 0.9 | 0.81 | 0.71 | 0.49 | 0.35 | 0.179 | 4.01 | 0.000787 | 0.9563 |
|  | **24** | 2 | 1.96 | 1.22 | 1.086 | 0.82 | 0.71 | 0.37 | 8.09 | 0.001249 | 0.9436 |
| **LBp5** | **4** | 0.03 | 0.3 | 0.2 | 0.4 | 0.3 | 0.3 | 0.1 | 1.33 | 0.727115  Non-significant | 0.0339 |
|  | **6** | 1.1 | 0.6 | 0.7 | 0.6 | 0.5 | 0.4 | 0.5 | 1.91 | 0.051238  Non-significant | 0.6545 |
|  | **12** | 1.2 | 0.8 | 0.9 | 0.9 | 0.6 | 0.5 | 0.1 | 4.00 | 0.010048 | 0.8416 |
|  | **24** | 1.8 | 2.1 | 1.3 | 1.9 | 0.8 | 0.8 | 0.2 | 8.06 | 0.024986 | 0.7545 |
| **Control** | **4** | 0.0001 | 0.2 | 0.5 | 0.4 | 1.74 | 2 | 3.1 | 1.47 | 0.005292 | 0.8836 |
|  | **6** | 0.7 | 0.5 | 0.4 | 0.7 | 0.5 | 7 | 1 | 2.73 | 0.373677  Non-significant | 0.2002 |
|  | **4** | 2 | 3 | 3.1 | 2 | 3 | 3.4 | 3.9 | 3.27 | 0.116347  Non-significant | 0.4996 |
|  | **6** | 2 | 3 | 3.1 | 2 | 3 | 3.4 | 3.9 | 7.48 | 0.116347  Non-significant | 0.4996 |

**Table S6: NaCl tolerance of *L. helveticus* isolates.**

| **Strain** | **Time**  **(hrs)** | **NaCl conc. (%)** | | | | | | | **Standard Deviation**  **SD** | **Pearson Correlation b/w Dose and Activity**  **(P=0.05)** | **R squared**  **(r^2^)** |
| --- | --- | --- | --- | --- | --- | --- | --- | --- | --- | --- | --- |
|  |  | **1** | **2** | **3** | **4** | **5** | **6** | **7** |  |  |  |
| **LBp1** | **4** | 0.46 | 0.43 | 0.36 | 0.32 | 0.22 | 0.18 | 0.19 | 1.30 | 0.00093 | 0.9518 |
|  | **6** | 1.22 | 0.8 | 0.64 | 0.54 | 0.33 | 0.19 | 0.14 | 1.95 | 0.00152 | 0.9388 |
|  | **12** | 1.26 | 0.88 | 0.74 | 0.57 | 0.33 | 0.25 | 0.16 | 4.04 | 0.000787 | 0.9553 |
|  | **24** | 2.09 | 1.69 | 1.14 | 0.93 | 0.77 | 0.46 | 0.32 | 8.13 | 0.000721 | 0.9571 |
| **LBp2** | **4** | 0.49 | 0.45 | 0.37 | 0.32 | 0.22 | 0.16 | 0.14 | 1.31 | 0.000121 | 0.9827 |
|  | **6** | 1.21 | 0.73 | 0.64 | 0.54 | 0.34 | 0.18 | 0.17 | 1.95 | 0.003125 | 0.9105 |
|  | **12** | 1.27 | 0.866 | 0.74 | 0.58 | 0.35 | 0.266 | 0.17 | 4.04 | 0.00093 | 0.9506 |
|  | **24** | 1.98 | 1.7 | 1.16 | 0.96 | 0.77 | 0.47 | 0.32 | 8.13 | 0.000336 | 0.9708 |
| **LBp3** | **4** | 1.33 | 1.23 | 1.06 | 0.88 | 0.8 | 0.7 | 0.56 | 1.11 | 0.000054 | 0.9892 |
|  | **6** | 1.8 | 1.72 | 1.56 | 1.26 | 0.9 | 0.8 | 0.6 | 1.74 | 0.000293 | 0.9734 |
|  | **12** | 1.86 | 1.73 | 1.63 | 1.43 | 1.06 | 0.8 | 0.7 | 3.80 | 0.000431 | 0.9669 |
|  | **24** | 2.1 | 1.99 | 1.9 | 1.8 | 1.3 | 0.8 | 0.8 | 7.96 | 0.003401 | 0.9065 |
| **LBp4** | **4** | 1.16 | 1.06 | 0.95 | 0.8 | 0.73 | 0.6 | 0.46 | 1.14 | 0.000013 | 0.9956 |
|  | **6** | 1.59 | 1.56 | 1.36 | 0.96 | 0.76 | 0.63 | 0.5 | 1.79 | 0.000596 | 0.962 |
|  | **12** | 1.6 | 1.5 | 1.4 | 1.23 | 0.95 | 0.6 | 0.56 | 3.86 | 0.000787 | 0.9551 |
|  | **24** | 1.66 | 1.85 | 1.73 | 1.73 | 1.19 | 0.6 | 0.6 | 8.02 | 0.020736 | 0.7744 |
| **LBp5** | **4** | 1.33 | 1.23 | 1.06 | 0.88 | 0.8 | 0.7 | 0.56 | 1.11 | .00015 | 0.9892 |
|  | **6** | 1.8 | 1.72 | 1.56 | 1.26 | 0.9 | 0.8 | 0.6 | 1.741 | 0.000293 | 0.9734 |
|  | **12** | 1.86 | 1.73 | 1.63 | 1.43 | 1.06 | 0.8 | 0.7 | 3.80 | 0.000431 | 0.9669 |
|  | **24** | 2.1 | 1.99 | 1.9 | 1.8 | 1.3 | 0.8 | 0.8 | 7.96 | 0.003401 | 0.9065 |
| **Control** | **4** | 0.0001 | 0.14 | 0.2 | 1 | 1.4 | 1.6 | 1 | 1.29 | 0.032063 | 0.7225 |
|  | **6** | 0.0009 | 0.1 | 0.3 | 0.7 | 1.5 | 1.8 | 2 | 1.95 | 0.000998 | 0.9489 |
|  | **4** | 0.0001 | 0.3 | 0.2 | 0.5 | 1.8 | 2.11 | 3 | 3.99 | 0.004898 | 0.8879 |
|  | **6** | 0.0001 | 0.2 | 0.5 | 0.4 | 1.74 | 2 | 3.1 | 8.15 | 0.005292 | 0.8836 |

**Table S7: Bile salts tolerance of *L. plantarum* isolates.**

| **Strains** | **Bile Salt Conc.** | **Time (hrs)** | | | | | | | **Standard Deviation**  **SD** | **Pearson Correlation b/w Dose and Activity (P=0.05)** | **R squared**  **r^2^** |
| --- | --- | --- | --- | --- | --- | --- | --- | --- | --- | --- | --- |
| **LBh1** |  | 0 | 2 | 4 | 6 | 10 | 12 | 24 |  |  |  |
|  | 0 | 0.06 | 0.25 | 0.61 | 0.74 | 0.98 | 1 | 1.21 | 0.41 | 0.000527 | 0.9628 |
|  | **0.05** | 0.06 | 0.24 | 0.56 | 0.64 | 0.81 | 0.98 | 0.98 | 0.35 | 0.000893 | 0.9516 |
|  | 0.1 | 0.06 | 0.23 | 0.42 | 0.53 | 0.64 | 0.72 | 0.75 | 0.25 | 0.000871 | 0.9522 |
|  | 0.3 | 0.06 | 0.22 | 0.33 | 0.5 | 0.59 | 0.69 | 0.7 | 0.24 | 0.000527 | 0.9628 |
|  | 0.6 | 0.06 | 0.06 | 0.06 | 0.06 | 0.06 | 0.06 | 0.06 | 0 | 0 | 0 |
|  | 1 | 0.06 | 0.06 | 0.06 | 0.06 | 0.06 | 0.06 | 0.06 | 0 | 0 | 0 |
| **LBh2** | 0 | 0.06 | 0.25 | 0.61 | 0.74 | 0.98 | 1 | 1.21 | 0.41 | 0.000527 | 0.9628 |
|  | **0.05** | 0.06 | 0.24 | 0.54 | 0.71 | 0.88 | 0.98 | 0.99 | 0.36 | 0.001301 | 0.9417 |
|  | 0.1 | 0.06 | 0.23 | 0.51 | 0.69 | 0.75 | 0.79 | 0.81 | 0.29 | 0.005629 | 0.88 |
|  | 0.3 | 0.06 | 0.22 | 0.51 | 0.64 | 0.71 | 0.73 | 0.78 | 0.27 | 0.005683 | 0.8795 |
|  | 0.6 | 0.06 | 0.06 | 0.06 | 0.06 | 0.06 | 0.06 | 0.06 | 0 | 0 | 0 |
|  | 1 | 0.06 | 0.06 | 0.06 | 0.06 | 0.06 | 0.06 | 0.06 | 0 | 0 | 0 |
| **LBh3** | 0 | 0.06 | 0.25 | 0.61 | 0.74 | 0.98 | 1 | 1.21 | 0.41 | 0.000527 | 0.9628 |
|  | **0.05** | 0.06 | 0.25 | 0.51 | 0.73 | 0.96 | 0.98 | 1.07 | 0.39 | 0.000879 | 0.952 |
|  | 0.1 | 0.06 | 0.23 | 0.49 | 0.7 | 0.77 | 0.79 | 0.8 | 0.29 | 0.006486 | 0.8714 |
|  | 0.3 | 0.06 | 0.22 | 0.49 | 0.68 | 0.73 | 0.72 | 0.78 | 0.28 | 0.006486 | 0.8608 |
|  | 0.6 | 0.06 | 0.06 | 0.06 | 0.06 | 0.06 | 0.06 | 0.06 | 0 | 0 | 0 |
|  | 1 | 0.06 | 0.06 | 0.06 | 0.06 | 0.06 | 0.06 | 0.06 | 0 | 0 | 0 |
| **LBh4** | 0 | 0.06 | 0.25 | 0.61 | 0.74 | 0.98 | 1 | 1.21 | 0.41 | 0.000527 | 0.9628 |
|  | **0.05** | 0.06 | 0.25 | 0.49 | 0.72 | 0.78 | 0.96 | 0.98 | 0.35 | 0.000754 | 0.9555 |
|  | 0.1 | 0.06 | 0.23 | 0.47 | 0.7 | 0.72 | 0.78 | 0.78 | 0.28 | 0.00639 | 0.8724 |
|  | 0.3 | 0.06 | 0.22 | 0.39 | 0.69 | 0.7 | 0.74 | 0.74 | 0.28 | 0.007301 | 0.8638 |
|  | 0.6 | 0.06 | 0.06 | 0.06 | 0.06 | 0.06 | 0.06 | 0.06 | 0 | 0 | 0 |
|  | 1 | 0.06 | 0.06 | 0.06 | 0.06 | 0.06 | 0.06 | 0.06 | 0 | 0 | 0 |
| **LBh5** | 0 | 0.06 | 0.25 | 0.61 | 0.74 | 0.98 | 1 | 1.21 | 0.41 | 0.000527 | 0.9628 |
|  | **0.05** | 0.06 | 0.25 | 0.51 | 0.73 | 0.96 | 0.98 | 1.07 | 0.39 | 0.000879 | 0.952 |
|  | 0.1 | 0.06 | 0.23 | 0.49 | 0.7 | 0.77 | 0.79 | 0.8 | 0.29 | 0.006486 | 0.8714 |
|  | 0.3 | 0.06 | 0.22 | 0.49 | 0.68 | 0.73 | 0.72 | 0.78 | 0.28 | 0.007631 | 0.8608 |
|  | 0.6 | 0.06 | 0.06 | 0.06 | 0.06 | 0.06 | 0.06 | 0.06 | 0 | 0 | 0 |
|  | 1 | 0.06 | 0.06 | 0.06 | 0.06 | 0.06 | 0.06 | 0.06 | 0 | 0 | 0 |
| **Control** | 0 | 0.0009 | 0.1 | 0.3 | 1.5 | 1.8 | 2 | 2.5 | 1.01 | 0.001445 | 0.9386 |
|  | **0.05** | 0.0009 | 0.1 | 0.3 | 1.5 | 1.8 | 2 | 2.5 | 1.01 | 0.001445 | 0.9386 |
|  | 0.1 | 0.0009 | 0.1 | 0.3 | 1.5 | 1.8 | 2 | 2.5 | 1.01 | 0.001445 | 0.9386 |
|  | 0.3 | 0.0009 | 0.1 | 0.3 | 1.5 | 1.8 | 2 | 2.5 | 1.01 | 0.001445 | 0.9386 |
|  | 0.6 | 0.0009 | 0.1 | 0.3 | 1.5 | 1.8 | 2 | 2.5 | 1.01 | 0.001445 | 0.9386 |
|  | 1 | 0.0009 | 0.1 | 0.3 | 1.5 | 1.8 | 2 | 2.5 | 1.01 | 0.001445 | 0.9386 |

**Table S8: Bile salts tolerance of *L. helveticus* isolates.**

| **Strains** | **Bile Salt Conc.** | **Time (hrs)** | | | | | | | **SD** | **Pearson Correlation b/w Dose and Activity (P=0.05)** | **R squared**  **r^2^** |
| --- | --- | --- | --- | --- | --- | --- | --- | --- | --- | --- | --- |
| **LBp1** |  | 0 | 2 | 4 | 6 | 10 | 12 | 24 |  |  |  |
|  | 0 | 0.08 | 0.28 | 0.64 | 0.76 | 1.1 | 1.2 | 1.25 | 0.45 | 0.000626 | 0.9594 |
|  | **0.05** | 0.08 | 0.27 | 0.57 | 0.69 | 0.85 | 1 | 1.1 | 0.37 | 0.000201 | 0.9769 |
|  | 0.1 | 0.08 | 0.25 | 0.44 | 0.59 | 0.7 | 0.78 | 0.78 | 0.27 | 0.001473 | 0.938 |
|  | 0.3 | 0.08 | 0.25 | 0.35 | 0.52 | 0.62 | 0.72 | 0.74 | 0.25 | 0.000377 | 0.9685 |
|  | 0.6 | 0.08 | 0.28 | 0.64 | 0.76 | 1.1 | 1.2 | 1.25 | 0 | 0 | 0 |
|  | 1 | 0.08 | 0.27 | 0.57 | 0.69 | 0.85 | 1 | 1.1 | 0 | 0 | 0 |
| **LBp2** | 0 | 0.08 | 0.28 | 0.64 | 0.76 | 1.1 | 1.2 | 1.25 | 0.45 | 0.000626 | 0.9594 |
|  | **0.05** | 0.08 | 0.27 | 0.56 | 0.73 | 0.9 | 1 | 1.1 | 0.38 | 0.000354 | 0.9694 |
|  | 0.1 | 0.08 | 0.25 | 0.53 | 0.71 | 0.78 | 0.81 | 0.83 | 0.29 | 0.00581 | 0.8782 |
|  | 0.3 | 0.08 | 0.24 | 0.53 | 0.68 | 0.74 | 0.77 | 0.8 | 0.28 | 0.006314 | 0.8731 |
|  | 0.6 | 0.08 | 0.08 | 0.08 | 0.08 | 0.08 | 0.08 | 0.08 | 0 | 0 | 0 |
|  | 1 | 0.08 | 0.08 | 0.08 | 0.08 | 0.08 | 0.08 | 0.08 | 0 | 0 | 0 |
| **LBp3** | 0 | 0.08 | 0.28 | 0.64 | 0.76 | 1.1 | 1.2 | 1.25 | 0.45 | 0.000626 | 0.9594 |
|  | **0.05** | 0.08 | 0.28 | 0.53 | 0.75 | 0.98 | 1 | 1.09 | 0.38 | 0.000857 | 0.9526 |
|  | 0.1 | 0.08 | 0.27 | 0.52 | 0.72 | 0.79 | 0.81 | 0.82 | 0.29 | 0.0067 | 0.8694 |
|  | 0.3 | 0.08 | 0.26 | 0.51 | 0.7 | 0.75 | 0.79 | 0.8 | 0.28 | 0.006181 | 0.8744 |
|  | 0.6 | 0.08 | 0.08 | 0.08 | 0.08 | 0.08 | 0.08 | 0.08 | 0 | 0 | 0 |
|  | 1 | 0.08 | 0.08 | 0.08 | 0.08 | 0.08 | 0.08 | 0.08 | 0 | 0 | 0 |
| **LBp4** | 0 | 0.08 | 0.28 | 0.64 | 0.76 | 1.1 | 1.2 | 1.25 | 0.45 | 0.000626 | 0.9594 |
|  | **0.05** | 0.08 | 0.28 | 0.51 | 0.74 | 0.8 | 0.98 | 1.1 | 0.37 | 0.000162 | 0.9793 |
|  | 0.1 | 0.08 | 0.26 | 0.49 | 0.72 | 0.75 | 0.8 | 0.81 | 0.29 | 0.005865 | 0.8776 |
|  | 0.3 | 0.08 | 0.25 | 0.4 | 0.71 | 0.72 | 0.76 | 0.76 | 0.28 | 0.007219 | 0.8645 |
|  | 0.6 | 0.08 | 0.08 | 0.08 | 0.08 | 0.08 | 0.08 | 0.08 | 0 | 0 | 0 |
|  | 1 | 0.08 | 0.08 | 0.08 | 0.08 | 0.08 | 0.08 | 0.08 | 0 | 0 | 0 |
| **LBp5** | 0 | 0.01 | 0.02 | 0.04 | 0.5 | 0.08 | 1.8 | 2 | 0.88 | 0.037733 | 0.7004 |
|  | **0.05** | 0.05 | 0.02 | 0.03 | 0.04 | 0.05 | 0.06 | 1.8 | 0.66 | 0.188242  Non-significant | 0.3856 |
|  | 0.1 | 0.01 | 0.02 | 0.05 | 0.08 | 0.09 | 0.09 | 0.09 | 0.034 | 0.007199 | 0.8647 |
|  | 0.3 | 0.08 | 0.26 | 0.51 | 0.7 | 0.75 | 0.79 | 0.8 | 0.28 | 0.006181 | 0.8744 |
|  | 0.6 | 0.08 | 0.08 | 0.08 | 0.08 | 0.08 | 0.08 | 0.08 | 0 | 0 | 0 |
|  | 1 | 0.08 | 0.08 | 0.08 | 0.08 | 0.08 | 0.08 | 0.08 | 0 | 0 | 0 |
| **Control** | 0 | 0.0009 | 0.1 | 0.3 | 1.5 | 1.8 | 2 | 2.5 | 1.01 | 0.001445 | 0.9386 |
|  | **0.05** | 0.0009 | 0.1 | 0.3 | 1.5 | 1.8 | 2 | 2.5 | 1.01 | 0.001445 | 0.9386 |
|  | 0.1 | 0.0009 | 0.1 | 0.3 | 1.5 | 1.8 | 2 | 2.5 | 1.01 | 0.001445 | 0.9386 |
|  | 0.3 | 0.0009 | 0.1 | 0.3 | 1.5 | 1.8 | 2 | 2.5 | 1.01 | 0.001445 | 0.9386 |
|  | 0.6 | 0.0009 | 0.1 | 0.3 | 1.5 | 1.8 | 2 | 2.5 | 1.01 | 0.001445 | 0.9386 |
|  | 1 | 0.0009 | 0.1 | 0.3 | 1.5 | 1.8 | 2 | 2.5 | 1.01 | 0.001445 | 0.9386 |

**Table S9: Antibiotic susceptibility of the isolates of *L. plantarum isolates*.**

| **Antibiotics** | **Zone of Inhibition in mm** | | | | | **Mean** | **Standard Deviation** |
| --- | --- | --- | --- | --- | --- | --- | --- |
|  | **LBp1** | **LBp2** | **LBp3** | **LBp4** | **LBp5** |  |  |
| **Amplillicin (2 mg/L)** | 7.3 | 6.2 | 5.5 | 1.8 | 17.2 | 7.6 | 5.75 |
| **Gentamycine (16 mg/L)** | 6.3 | 9.4 | 0 | 12.6 | 0 | 5.66 | 5.63 |
| **Kanamycin (64mg/L)** | 38.1 | 21.5 | 0 | 22.9 | 0 | 16.5 | 16.41 |
| **Erythromycin (1mg/L)** | 0 | 0 | 0 | 0 | 2.6 | 0.52 | 1.16 |
| **Clindamycin (4mg/L)** | 0 | 0 | 0 | 0 | 0 | 0 | 0.00 |
| **Tetracycline (32mg/L)** | 19.9 | 26 | 0 | 10.5 | 0 | 11.28 | 11.68 |
| **Chloramphenicol (8mg/L)** | 23.8 | 16.1 | 19.5 | 3.4 | 11.4 | 14.84 | 7.85 |

**Table S10: Antibiotic susceptibility of the isolates of *L. helveticus isolates*.**

| **Antibiotics** | **Zone of Inhibition in mm** | | | | | **Mean** | **Standard Deviation** |
| --- | --- | --- | --- | --- | --- | --- | --- |
|  | **LBh1** | **LBh2** | **LBh3** | **LBh4** | **LBh5** |  |  |
| **Ampillicin (2 mg)** | 2.8 | 5.9 | 10.7 | 7.2 | 7.2 | 6.76 | 2.54 |
| **Vancomycine(2 mg)** | 3.3 | 10.5 | 0 | 5.4 | 0 | 3.84 | 3.91 |
| **Gentamycin19 (16 mg)** | 15.1 | 25.7 | 0 | 19.4 | 0 | 12.04 | 10.39 |
| **Kanamycin (16 mg)** | 8.6 | 15.4 | 13.4 | 16.2 | 7.8 | 12.28 | 3.46 |
| **Strepto mycin (16mg/L)** | 2.5 | 7.4 | 0 | 5.9 | 0 | 3.16 | 3.03 |
| **Erythromycin (1mg/L)** | 5.6 | 4.4 | 0 | 5.6 | 0 | 3.12 | 2.58 |
| **Clindamycin (4mg/L)** | 0 | 3.2 | 0 | 0 | 0 | 0.64 | 1.28 |

**
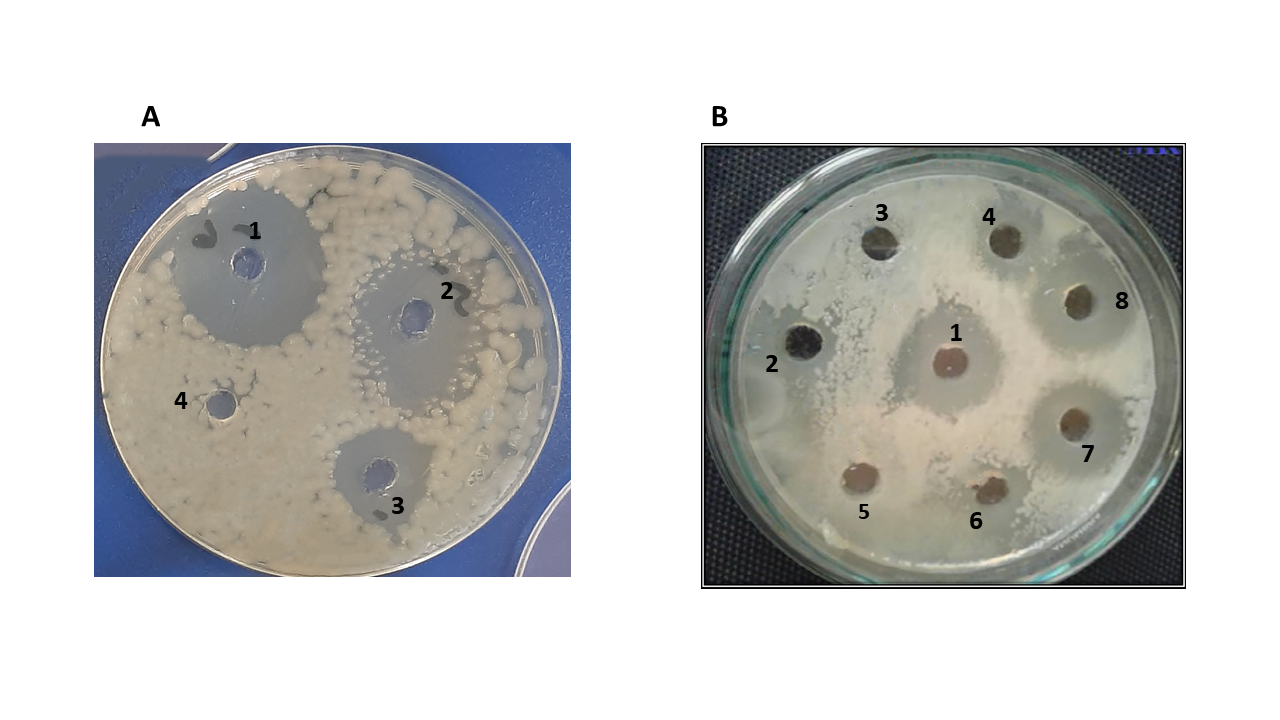
**

**Figure S1-Antibiotic susceptibility tests against *L.palantarum* and *L.helveticus* strains - (A)** LBp1 against antibiotics; Well 1 containes kanamycin (38.3 mg/L), Well 2 contains for chloroamphenicol (23.8 mg/L) ,Well 3 contains tetracylcine (32 mg/L) ,4 stands for the control **(B)** LBh4 against antibiotics ,Well 1 contains gentamycin(19.4 mg/L) ,Well 2 contains the vancomycin (2mg/L), Well 3 contains control ,Well 4 contains erthyromycin (1mg/L), Well 5 stands for the clindamycin(4 mg/L),Well 6 contains for the streptomycin (16mg/L),Well 7 contains amplliicin (2 mg/L) ,Well 8 contains kanamycin(16mg/L),

**Table S11:. Antibacterial activity of *L.plantarum* strains against foodborne pathogens**

| **Name of Microbes** | **Strains** | | | | | **Standard Deviation**  **SD** | **Pearson Correlation b/w Dose and Activity (P=0.05)** | **R squared**  **(r^2^)** |
| --- | --- | --- | --- | --- | --- | --- | --- | --- |
|  | **LBp1** | **Lbp2** | **LBp3** | **LBp4** | **LBp5** |  |  |  |
| **Gram-negative bacteria** | | | | | | | | |
| ***Klebsiella pneumonia*** | 5.6 | 5.7 | 5.8 | 5.9 | 4.9 | 0.39 | 0.337608  Non-significant | 0.2293 |
| ***Pseudomonas aerogenosa*** | 6.1 | 6.2 | 6.3 | 6.4 | 5.6 | 0.31 | 0.424462  Non-significant | 0.1649 |
| ***Actinobacter baumannii*** | 20.35 | 20.45 | 21 | 21.1 | 22.9 | 1.02 | 0.018915 | 0.7841 |
| ***Salmonella paratyphi*** | 16.6 | 16.7 | 16.8 | 16.17 | 17.8 | 0.60 | 0.321775  Non-significant | 0.2419 |
| ***E. coli*** | 11.2 | 11.4 | 11.6 | 11.7 | 12.7 | 0.58 | 0.01484 | 0.8078 |
| **Gram-positive bacteria** | | | | | | | | |
| ***MRSA*** | 14.5 | 14.6 | 14.5 | 14.9 | 15.9 | 0.59 | 0.042685 | 0.6826 |
| ***Staphylococcus aureus*** | 21.1 | 21.2 | 21.3 | 21.9 | 22.9 | 0.74 | 0.012598 | 0.8225 |
| ***Streptococcus pyogenes*** | 5.35 | 5.65 | 5.85 | 5.95 | 6.8 | 0.54 | 0.00672 | 0.8692 |
| ***Bacillus subtilis*** | 12.35 | 12.45 | 12.55 | 12.65 | 13.2 | 0.33 | .013464 | 0.8167 |
| ***Enterococcus faesium*** | 16.5 | 16.65 | 16.7 | 16.9 | 17.8 | 0.51 | 0.02407 | 0.7578 |
| **Control** | 0 | 0 | 0 | 0 | 0 | 0 | 0 | 0 |

**
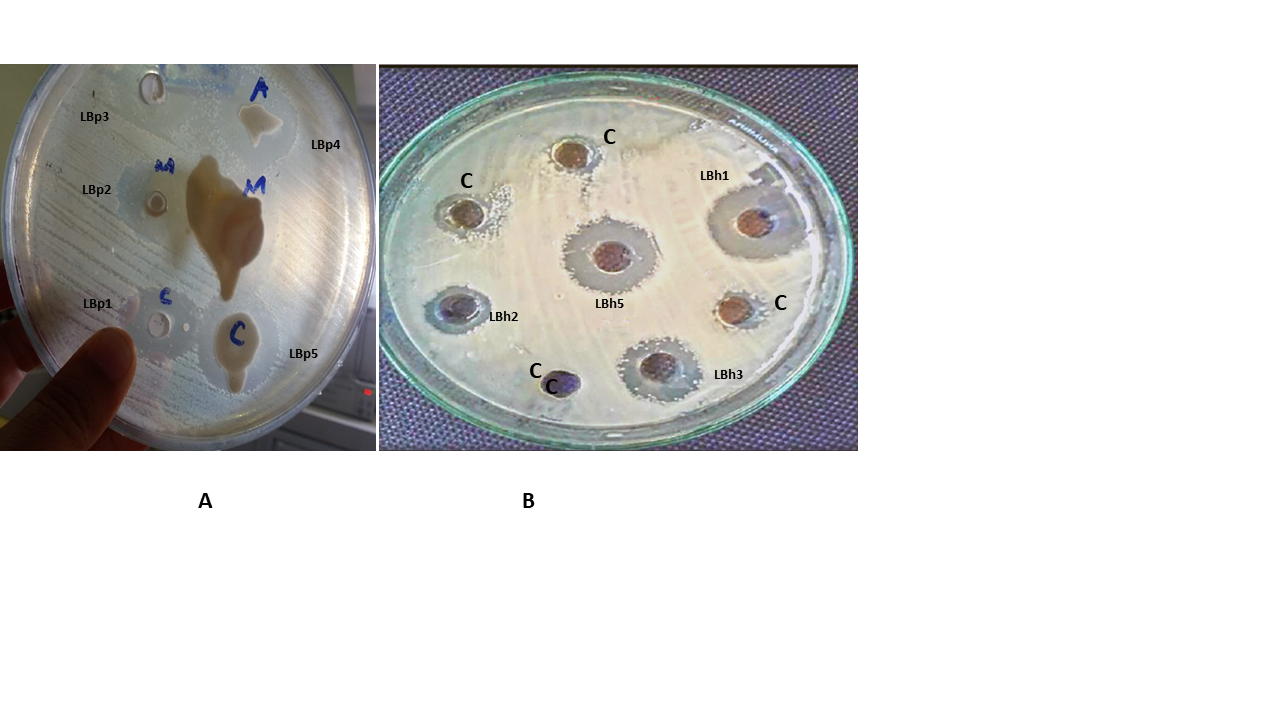
**

**Figure S2-Antibacterial of *L.palantarum* and *L.helveticus* strains -(A)** ***L.palantarum strains* activity against *Staphylococcus aureus*  (B) *L.helveticus* strains activity against *Staphylococcus aureus***

**Table S12:. Antibacterial activity of *L. helveticus*  isolates against foodborne pathogens**

| **Name of microbes** |  | |  | **Strains** |  | |  | **Standard**  **Deviation SD** | **Pearson Correlation b/w**  **Dose and Activity (P=0.05)** | **R squared**  **(r^2^)** |
| --- | --- | --- | --- | --- | --- | --- | --- | --- | --- | --- |
|  | **LBh1** | | **LBh2** | **LBh3** | **LBh4** | | **LBh5** |  |  |  |
| **Gram-negative bacteria** | | | | | | | | | | |
| ***Klebsiella pneumonia*** | 4.6 | | 4.7 | 4.9 | 4.8 | | 5 | 0.15 | .0145 | 0.81 |
| ***Pseudomonas aerogenosa*** | 3.1 | | 3.2 | 4.8 | 3.4 | | 4.9 | 0.89 | 0.142911  Non-significant | 0.4529 |
| ***Actinobacter baumannii*** | 18.35 | | 18.45 | 19.1 | 14.8 | | 15.2 | 2.01 | 0.067276  Non-significant | 0.6092 |
| ***Salmonella paratyphi*** | 14.6 | | 14.7 | 14.8 | 14.7 | | 15.9 | 0.54 | 0.079805  Non-significant | 0.5768 |
| ***E. coli*** | 9.2 | | 9.4 | 9.8 | 9.7 | | 10.1 | 0.35 | 0.00417 | 0.8964 |
| **Gram-positive bacteria** | | | | | | | | | | |
| ***MRSA*** | | 12.5 | 12.6 | 12.9 | 12.5 | | 13.1 | 0.26 | .163875  Non-significant | 0.4202 |
| ***Staphylococcus aureus*** | | 18.1 | 18.2 | 18.9 | 18.3 | 19.5 | | 0.59 | 0.070182  Non-significant | 0.6008 |
| ***Streptococcus pyogenes*** | | 3.35 | 3.65 | 3.95 | 3.85 | 4.1 | | 0.29 | 0.008292 | 0.8551 |
| ***Bacillus subtilis*** | | 10.34 | 10.45 | 10.66 | 10.56 | 11.2 | | 0.33 | 0.025693 | 0.7501 |
| ***Enterococcus faecium*** | | 14.5 | 14.65 | 14.9 | 14.7 | 11.2 | | 1.56 | 0.152902  Non-significant | 0.4372 |
| **Control** | | 0 | 0 | 0 | 0 | 0 | | 0 | 0 | 0 |

1

2

**Table S13 -Response to the stomach-deudonal stimulus of *L. plantarum* and *L. helveticus* isolates.**

| **Strains** | **TIME (hrs)** | | | | | | | | **Standard Deviation**  **SD** | **Pearson’s correlation**  **P value** | **R squared**  **r^2^** |
| --- | --- | --- | --- | --- | --- | --- | --- | --- | --- | --- | --- |
|  | 0 | 2 | 4 | 6 | 8 | 10 | 12 | 14 |  |  |  |
| **LBh1** | 0.0009 | 0.1 | 0.4 | 0.4 | 0.7 | 0.9 | 1.8 | 1.9 | 0.72 | 0.003732 | 0.9019 |
| **LBh2** | 0.0001 | 0.3 | 0.5 | 0.5 | 0.9 | 0.9 | 0.9 | 0.9 | 0.34 | 0.008488 | 0.8534 |
| **LBh3** | 0.0001 | 0.2 | 0.6 | 0.6 | 0.8 | 0.9 | 1 | 2 | 0.60 | 0.00907 | 0.8486 |
| **LBh4** | 0.0009 | 0.1 | 0.7 | 0.7 | 0.8 | 0.9 | 1.9 | 2.5 | 0.85 | 0.006977 | 0.8668 |
| **LBh5** | 0.0001 | 0.3 | 0.6 | 0.6 | 0.5 | 0.5 | 0.5 | 0.5 | 0.19 | 0.208865  Non-significant | 0.3589 |
| **Control** | 0.0009 | o.1 | 0.3 | 0.7 | 1.65 | 1.7 | 1.6 | 1.4 | 0.70 | 0.017547 | 0.7917 |
| **LBP1** | 0.0009 | 0.1 | 0.3 | 0.7 | 0.7 | 0.9 | 1.8 | 1.9 | 0.71 | 0.002746 | 0.9157 |
| **LBP2** | 0.0001 | 0.3 | 0.2 | 0.5 | 0.9 | 0.9 | 0.9 | 0.9 | 0.37 | 0.008889 | 0.8501 |
| **LBP3** | 0.0001 | 0.2 | 0.5 | 0.4 | 0.8 | 0.9 | 1 | 2 | 0.62 | 0.009092 | 0.8484 |
| **LBP4** | 0.0009 | 0.1 | 0.3 | 0.7 | 0.8 | 0.9 | 1.9 | 2.5 | 0.87 | 0.005102 | 0.8857 |
| **LBP5** | 0.0001 | 0.3 | 0.2 | 0.5 | 0.5 | 0.5 | 0.5 | 0.5 | 0.19 | 0.043258 | 0.6806 |
| **Control** | 0.0009 | 0.1 | 0.3 | 0.7 | 1.5 | 1.8 | 2 | 2.5 | 0.95 | 0.000441 | 0.9659 |
